# Revision of the genus *Splanchospora* (*Pleosporales*, *Neohendersoniaceae*)

**DOI:** 10.3897/imafungus.17.179372

**Published:** 2026-03-09

**Authors:** Ryuichi Yoshioka, Hermann Voglmayr, Akira Hashimoto, Misato Matsumura, Yoshihiro Kudo, Kazuaki Tanaka

**Affiliations:** 1 Faculty of Agriculture and Life Science, Hirosaki University, 3 Bunkyo-cho, Hirosaki, Aomori 036-8561, Japan Microbe Division / Japan Collection of Microorganisms RIKEN BioResource Research Center Ibaraki Japan; 2 The United Graduate School of Agricultural Sciences, Iwate University, 18-8 Ueda 3 chome, Morioka, Iwate 020-8550, Japan Faculty of Agriculture and Life Science, Hirosaki University Hirosaki Japan; 3 Department of Botany and Biodiversity Research, University of Vienna, Rennweg 14, 1030 Wien, Austria Department of Botany and Biodiversity Research, University of Vienna Wien Austria; 4 Current address: Microbe Division / Japan Collection of Microorganisms RIKEN BioResource Research Center, 3-1-1 Koyadai, Tsukuba, Ibaraki 305-0074, Japan The United Graduate School of Agricultural Sciences, Iwate University Morioka Japan

**Keywords:** *
Ascomycota
*, cryptic species, cultural study, four novel taxa, host preference, phylogeny, taxonomy, *
Tilia
*

## Abstract

A completely asexual morph of *Splanchospora* is revealed for the first time. This genus is characterised by clavate-ellipsoid, thick-walled ascospores strongly constricted at the submedian septum, the presence of paraphyses in the conidiomata, and globose to ellipsoid, 1-celled conidia. Through phylogenetic analyses based on the small subunit (SSU) and large subunit (LSU) of the nuclear ribosomal DNA, the nuclear ribosomal internal transcribed spacer (ITS) region, the second largest RNA polymerase II subunit (*RPB2*) gene, and the translation elongation factor 1-alpha (*TEF1*) gene, the genus is clarified to belong to the family *Neohendersoniaceae* (*Pleosporales*, *Dothideomycetes*). The type species, *Splanchospora ampullacea*, is neotypified and found to be a species complex. Because of this, we describe four new *Splanchospora* species, *S. fulviconidia***sp. nov**., *S. microspora***sp. nov**., *S. oblongiconidia***sp. nov**., and *S. pseudomicrospora***sp. nov**. from the twigs of lindens in Japan. All *Splanchospora* species examined show slight differences in conidial morphology, such as size, shape, and pigmentation. Species-level analyses suggest that this genus, previously considered to be monotypic, in fact harbours a remarkable diversity of species existing on lindens worldwide.

## Introduction

The monotypic genus *Splanchospora* Lar.N. Vassiljeva was established in 1998 to rearrange *Splanchnonema ampullaceum* (Basionym: *Sphaeria ampullacea* Pers.), inhabiting twigs of *Tilia* spp. L. native to Europe. The genus is characterised solely by 2-celled ascospores with a strongly submedian primary septum (Vasilyeva 1998; Tanaka et al. 2005). Although a detailed description of the sexual morph of the type species, *Splanchospora ampullacea*, was provided by Barr (1982) and Cannon and Minter (2014), additional characterisation of the genus on the basis of the asexual morph has not yet been performed. Tonolo (1956) carried out a culture study of *Massariella curreyi*, a synonym of *S. ampullacea*, and observed a phoma-type pycnidial asexual morph. However, Barr (1982) pointed out that this may be a spermatial state. Due to the ambiguous morphological circumscription of *Splanchospora*, Cannon and Minter (2014) suggested that this species should be retained in the genus *Splanchnonema* rather than *Splanchospora*. According to Vasilyeva (1998), *Splanchospora* belongs to *Massariaceae*; however, this classification could so far not be verified by phylogenetic analysis owing to the absence of *S. ampullacea* sequence data. Therefore, to date, the genus remains as *Ascomycota* genus *incertae sedis* (Hyde et al. 2024).

Peck (1894) described *Massariella curreyi* var. americana from *Tilia americana* L. in the USA as being different from the typical *M. curreyi* found in Europe. However, Barr (1982) subsequently re-examined the holotype of this North American variety and found that it did not differ sufficiently, on the basis of the sexual morph alone, from its European counterpart and synonymised it with *M. curreyi*. *Splanchospora ampullacea* is considered a semi-cosmopolitan species which colonises various linden species and has even been reported in the Russian Far East (Vasilyeva 1998).

In our ongoing taxonomic study of dothideomycetous fungi (e.g., Yoshioka et al. 2025), several fresh specimens of sexual morphs of *Splanchospora* spp. were collected from the twigs of lindens. In most of these specimens, coelomycetous asexual morphs were consistently observed to be associated with the sexual morphs. Under culture conditions, single ascospore isolates formed asexual morphs matching those observed on the original specimens. In this study, we aimed to revise the generic concept of *Splanchospora* by incorporating information from the asexual morphs, to clarify the phylogenetic position of the genus, and to identify the species found in Japan.

## Materials and methods

### Specimens and isolates

Twenty-eight fresh fungal specimens were collected from dead twigs of *Tilia* spp. (*Malvaceae*), *Carpinus cordata* Blume, and *Ostrya japonica* Sarg. (both *Betulaceae*) in Japan and Europe from 2006 to 2024. Twenty-six of these specimens were collected from linden trees, most planted in botanical gardens or parks. All specimens were deposited at the Fungaria of Hirosaki University (HHUF) and/or the Department of Botany and Biodiversity Research, University of Vienna (WU). Single-spore isolates were obtained from the fresh specimens, and representative isolates were deposited at the NARO Genebank (MAFF), Tsukuba, Japan; or the Westerdijk Fungal Biodiversity Institute (the CBS Culture Collection), Utrecht, The Netherlands. Additionally, a strain of *S. ampullacea* (CBS 116578) was purchased from the Westerdijk Fungal Biodiversity Institute for comparative analysis. For detailed information on these specimens and isolates, see Suppl. material 1: table SS1.

Colony characteristics were recorded from growth on potato dextrose agar (PDA) at 20 °C in the dark for 2 weeks. Colony colours were determined by referring to Rayner (1970). Several pieces of mycelial agar were placed on water agar containing sterilised banana leaves, Japanese beech twigs, and rice straws to observe sporulation *in vitro*. After the mycelia had colonised the substrates at 20 °C for 2 weeks, the plates were incubated at 20 °C under blacklight blue illumination for 2–4 weeks to induce sporulation.

### Morphological observations and comparison of spore sizes

Macroscopic morphological observations were made using an Olympus SZX10 stereomicroscope (Olympus, Tokyo, Japan). Slide preparations were typically mounted in distilled water. Shear’s mounting medium was used to mount sections of ascomata/conidiomata. Morphological characteristics were observed using an Olympus BX 51 or 53 biological microscope equipped with an Olympus DP21 digital microscopy camera (Olympus), a Zeiss Axio Imager.A1 compound microscope equipped with a Zeiss Axiocam 506 colour digital camera (Zeiss, Jena, Germany), or a Leitz Ortholux microscope equipped with a Nikon 995 colour digital camera. The position of the primary septum in the ascospores was determined using a decimal system, expressed as the ratio of the upper hemisphere length to the total ascospore length according to the method described by Shoemaker (1984). Means ± standard deviation (SD) were calculated for sizes of asci, ascospores, conidiogenous cells, and conidia; length of ascus stalk; l/w ratios of ascospores and conidia; and ratios of the upper hemisphere length to the total ascospore length. Scatter plots of the measured spore sizes were created to confirm the morphological differences.

### DNA extraction, PCR amplification, and sequencing

DNA was extracted from the cultures using the ISOPLANT II Kit (Nippon Gene, Tokyo, Japan) according to the manufacturer’s instructions, the DNeasy Plant Mini Kit (QIAgen GmbH, Hilden, Germany) according to Voglmayr and Jaklitsch (2011) and Jaklitsch et al. (2012), or the GeneJET Plant Genomic DNA Purification Kit (Thermo Fisher Scientific Inc., Waltham, MA, USA).

The following loci were amplified and sequenced: the small subunit (SSU) and large subunit (LSU) of the nuclear ribosomal DNA, the internal transcribed spacer (ITS) region, the second largest RNA polymerase II subunit (*RPB2*) gene, and the translation elongation factor 1-alpha (*TEF1*) gene. The primers and annealing temperatures used for the polymerase chain reaction (PCR) are listed in Suppl. material 1: table SS2. Most PCR products were purified using the FastGene Gel/PCR Extraction Kit (Nippon Gene), according to the manufacturer’s instructions and sequenced using the analysis services of SolGent (Daejeon, Korea). The PCR products from strains L20, L39, L104, L179, and L181 were purified using enzymatic PCR clean-up (Werle et al. 1994), as described by Voglmayr and Jaklitsch (2008). The DNA from the five strains was cycle-sequenced using the ABI PRISM Big Dye Terminator Cycle Sequencing Ready Reaction Kit v.3.1 (Applied Biosystems; Warrington, UK). Sequencing was performed using an automated DNA sequencer (ABI 3730xl Genetic Analyzer, Applied Biosystems). The newly generated sequences were deposited into the GenBank database (Table 1).

**Table 1.** Taxa information and GenBank accession numbers of the sequences used in this study.

Taxon	Culture/voucher no.	Type^†^	GenBank accession no.					References
			SSU	ITS	LSU	*RPB2*	*TEF1*	
*Acrocalymma medicaginis*	MFLU 18-2258		OR134384	OR224990	OP612529	OR146903	OR140362	Zhang et al. 2023
*Aigialus parvus*	PUFD45^‡^		MK026763	MK028710	MK026761	MN520612	MN520611	Hongsanan et al. 2020
‘*Amarenographium*’ *solium*	MFLUCC 12-0087	H	JX181943	–	JX181942	–	–	Hodhod et al. 2012
*Anastomitrabeculia didymospora*	MFLUCC 11-0200		ON077074	ON077080	ON077069	ON075067	ON075063	Phookamsak et al. 2022
*Ascocylindrica marina*	MD6011^‡^	H	KT252907	–	KT252905	–	–	Ariyawansa et al. 2015
*Brevicollum hyalosporum*	MAFF 243400	H	LC271236	LC271242	LC271239	LC271249	LC271245	Tanaka et al. 2017
*Brevicollum versicolor*	MAFF 246251	H	LC271237	LC271243	LC271240	LC271250	LC271246	Tanaka et al. 2017
*Camarographium clematidis*	ZHKUCC 23-0039		PP809706	OR825374	PP809726	–	PP812257	Du et al. 2025
*Crassiparies octosporus*	MFLUCC 18-0304a	H	–	OL782147	OL782065	–	OL875105	Senwanna et al. 2021
*Crassiparies quadrisporus*	MAFF 245408	H	LC100017	LC100020	LC100025	LC271251	LC271247	Li et al. 2016; Tanaka et al. 2017
*Crassiparies yunnanensis*	KUMCC 21-0215	H	OK564663	OK564664	OK564661	OK562422	OK562423	Lu et al. 2022
*Delitschia chaetomioides*	CBS 147251		MW209047	MW209042	MW209067	MW238833	MW238837	Pintye and Knapp 2021
*Didymella exigua*	CBS 183.55	N	EU754056	EF192139	EU754155	EU874850	genome^¶^	Zuccaro et al. 2008; Chilvers et al. 2009; de Gruyter et al. 2009; Haridas et al. 2020
*Halojulella avicenniae*	BCC 20173		GU371830	–	GU371822	GU371786	GU371815	Schoch et al. 2009
*Helminthosporium velutinum*	CBS 139923	E	KY984432	KY984352	KY984352	KY984413	KY984463	Voglmayr and Jaklitsch 2017
*Hysterium angustatum*	KUMCC 21-0213		OK442653	OK482567	OK482568	OK506219	OK398120	Manawasinghe et al. 2022
*Hysterobrevium constrictum*	KUMCC 21-0212		OK442652	OK482565	OK482566	OK506220	OK398119	Manawasinghe et al. 2022
*Leptosphaeria doliolum*	CBS 505.75	I^§^	GU296159	JF740205	GU301827	KY064035	GU349069	Schoch et al. 2009; de Gruyter et al. 2013; Marin-Felix et al. 2017
*Lophiostoma macrostomum*	MAFF 239447		AB521732	AB433276	AB433274	JN993493	LC001753	Tanaka and Hosoya 2008; Hirayama et al. 2010; Schoch et al. 2012; Thambugala et al. 2015
*Lophiotrema nucula*	CBS 627.86		AB618703	LC194497	AB619021	LC194465	LC194410	Hirayama and Tanaka 2011; Hashimoto et al. 2017b
*Macrodiplodiopsis desmazieri*	CBS 140062	E	–	KR873240	KR873272	–	–	Crous et al. 2015
*Massaria inquinans*	M19		HQ599444	HQ599402	HQ599402	HQ599460	HQ599342	Voglmayr and Jaklitsch 2011
*Massaria vomitoria*	M13		HQ599440	HQ599437	HQ599437	HQ599466	HQ599375	Voglmayr and Jaklitsch 2011
*Massarina eburnea*	CBS 473.64		GU296170	AF383959	GU301840	GU371732	GU349040	Liew et al. 2002; Schoch et al. 2009
*Medicopsis romeroi*	CBS 252.60	H	EU754108	KF366446	EU754207	KF015708	KF015678	de Gruyter et al. 2009; Ahmed et al. 2014
*Melanomma japonicum*	CBS 142905	H	LC203293	LC203321	LC203339	LC203395	LC203367	Hashimoto et al. 2017a
*Muriformispora magnoliae*	MFLUCC 19-0036	H	OL824795	OM212459	OL813499	ON502385	ON303277	de Silva et al. 2022
*Muriformispora thailandica*	CMUB 40048	H	PQ408650	PQ409508	PQ408647	PQ423762	PQ407637	Wu et al. 2024
*Neobrevicollum biancaeae*	CGNCC 3.25420	H	OR754093	OR754085	OR754078	OR855453	OR855446	Lu et al. 2024
*Neobrevicollum oleae*	UESTCC 23.0145		OR754091	OR754083	OR754076	OR855458	OR855451	Lu et al. 2024
*Neohendersonia kickxii*	CBS 112403	E	–	KX820255	KX820266	–	–	Giraldo et al. 2017
*Neohendersonia tongrenensis*	HKAS 136895	H	–	PQ325268	PQ578302	–	–	Sun et al. 2025
*Neomedicopsis chiangmaiensis*	MFLUCC 17-2457	H	MG873483	MG873485	MG873481	–	–	Hyde et al. 2018
*Neomedicopsis prunicola*	CBS 145031	H	–	MK442603	MK442539	MK442670	–	Crous et al. 2019
*Omania hydei*	SQUCC 3026	H	MW077162	MW077146	MW077155	–	MW075772	Maharachchikumbura et al. 2021
*Paralentithecium aquaticum*	CBS 123099	H	GU296156	MH863276	GU301823	GU371789	GU349068	Schoch et al. 2009; Vu et al. 2019
*Phaeosphaeria oryzae*	CBS 110110	E	GQ387530	KF251186	GQ387591	ON419520	ON419509	de Gruyter et al. 2010; Quaedvlieg et al. 2013; Ashrafi et al. 2023
*Prosthemium betulinum*	CBS 279.74		DQ678027	AB554089	DQ678078	DQ677976	DQ677923	Schoch et al. 2006b; Tanaka et al. 2010
*Pseudosplanchnonema phorcioides*	CBS 122935		KY984434	KY984360	KY984360	KY984418	KY984467	Voglmayr and Jaklitsch 2017
*Splanchnonema pupula*	MFLU 14-0807		–	KP659196	KP659197	–	–	Liu et al. 2015
*Splanchospora ampullacea*	CBS 116578		**LC895714**	**LC895733**	**LC895757**	**LC895795**	**LC895776**	This study
	L20		**PX559689**	**PX559684**	**PX559684**	**PX559185**	**PX559190**	This study
	L39		–	**PX559685**	**PX559685**	**PX559186**	**PX559191**	This study
	L104		–	**PX559686**	**PX559686**	**PX559187**	**PX559192**	This study
	L179	N	–	**PX559687**	**PX559687**	**PX559188**	**PX559193**	This study
*Splanchospora fulviconidia*	RY 69	P	**LC895727**	**LC895748**	**LC895770**	**LC895808**	**LC895789**	This study
	RY 71	P	**LC895728**	**LC895749**	**LC895771**	**LC895809**	**LC895790**	This study
	RY 73	P	**LC895729**	**LC895750**	**LC895772**	**LC895810**	**LC895791**	This study
	RY 91	H	**LC895730**	**LC895751**	**LC895773**	**LC895811**	**LC895792**	This study
	RY 92	P	**LC895731**	**LC895752**	**LC895774**	**LC895812**	**LC895793**	This study
	RY 112	P	**LC895732**	**LC895753**	**LC895775**	**LC895813**	**LC895794**	This study
	RY 152	P	–	**LC895754**	–	–	–	This study
	RY 153	P	–	**LC895755**	–	–	–	This study
	RY 161	P	–	**LC895756**	–	–	–	This study
*Splanchospora microspora*	KT 2905	P	**LC895715**	**LC895734**	**LC895758**	**LC895796**	**LC895777**	This study
	KT 2906	H	**LC895716**	**LC895735**	**LC895759**	**LC895797**	**LC895778**	This study
*Splanchospora oblongiconidia*	AH 478	P	**LC895724**	**LC895745**	**LC895767**	**LC895805**	**LC895786**	This study
	KT 3610	P	**LC895725**	**LC895746**	**LC895768**	**LC895806**	**LC895787**	This study
	RY 83	H	**LC895726**	**LC895747**	**LC895769**	**LC895807**	**LC895788**	This study
*Splanchospora pseudomicrospora*	KT 3680	P	**LC895717**	**LC895736**	**LC895760**	**LC895798**	**LC895779**	This study
	KT 3681	P	**LC895718**	**LC895737**	**LC895761**	**LC895799**	**LC895780**	This study
	KT 4246	H	**LC895719**	**LC895738**	**LC895762**	**LC895800**	**LC895781**	This study
	KT 4247	P	**LC895720**	**LC895739**	**LC895763**	**LC895801**	**LC895782**	This study
	KT 4283	P	**LC895721**	**LC895740**	**LC895764**	**LC895802**	**LC895783**	This study
	KT 4324	P	**LC895722**	**LC895741**	**LC895765**	**LC895803**	**LC895784**	This study
	RY 50	P	**LC895723**	**LC895742**	**LC895766**	**LC895804**	**LC895785**	This study
	RY 154	P	–	**LC895743**	–	–	–	This study
	RY 158	P	–	**LC895744**	–	–	–	This study
*Splanchospora* sp.	L181		–	**PX559688**	**PX559688**	**PX559189**	**PX559194**	This study
*Stemphylium vesicarium*	CBS 191.86	T^|^	DQ247812	DQ491516	DQ247804	DQ247794	DQ471090	Schoch et al. 2006a; Spatafora et al. 2006; James et al. 2006
*Trematosphaeria pertusa*	CBS 122368	E	FJ201991	KF015668	FJ201990	FJ795476	KF015701	Zhang et al. 2008; Zhang et al. 2009; Ahmed et al. 2014

Bold letters indicate sequences generated in this study. † E = ex-epitype, H = ex-holotype, I = ex-isotype, N = ex-neotype, P = ex-paratype, T = ex-type. ‡ Strain designation from GenBank. § Isotype of *Phoma hoehnelii* subsp. hoehnelii. | Type of *Stemphylium herbarum*. ¶ Sequence retrieved from genome deposited at JGI-DOE (http://genome.jgi.doe.gov/).

### Phylogenetic and barcode analyses

The first phylogenetic analysis of the SSU, ITS, LSU, *RPB2*, and *TEF1* sequences from 64 ingroup strains/specimens of *Pleosporales* was performed to clarify the familial placement of *Splanchospora*. Except for those of *Splanchospora*, the other 40 members of *Pleosporales* were retrieved from the GenBank database (see Table 1 for details on the sources of the sequences). Two members of *Hysteriales* were selected as outgroups. For the second phylogenetic analysis, single-gene trees based on the ITS, *RPB2*, and *TEF1* sequences as well as a combined tree of these three loci were generated to assess the species boundaries of the 24 strains (29 strains in the ITS tree) within *Splanchospora*. The FASTA-format sequence alignments used in these analyses are available in the Suppl. materials 1–9.

The multiple sequence alignment program MUSCLE, implemented in MEGA v.7.0 (Kumar et al. 2016), was used to align the SSU, ITS, LSU, *RPB2*, and *TEF1* sequences. Maximum-likelihood (ML) and Bayesian methods were used for the phylogenetic analyses. Kakusan4 software (Tanabe 2011) was used to estimate the optimum substitution models for each dataset, using the Akaike information criterion (Akaike 1974) or the corrected Akaike information criterion (AICc; Sugiura 1978) for the ML analysis and the Bayesian information criterion (BIC; Schwarz 1978) for the Bayesian analysis. The TreeFinder Mar 2011 program (http://www.treefinder.de) was used for the ML analysis based on the models selected using the AICc4 parameter. The ML bootstrap support (MLBS) values were obtained using 1,000 bootstrap replicates. The Bayesian analysis program MrBayes v.3.2.6 (Ronquist et al. 2012) was executed using substitution models selected on the basis of the BIC4 parameter. Two simultaneous and independent Metropolis-coupled Markov chain Monte Carlo (MCMCMC) runs were performed for 3,000,000, 2,000,000, and 1,500,000 generations for the first, second combined, and second single-gene analyses, respectively, with trees sampled every 1,000 generations. The convergence of the MCMCMC procedure was assessed on the basis of the effective sample size scores (all >100) using MrBayes and Tracer v.1.7.2 (Rambaut et al. 2018). The first 25% of trees were discarded during the burn-in phase, and the remainder were used to calculate the 50% majority-rule trees and determine the Bayesian posterior probability (BPP) values for individual branches. Statistical support was defined as full (100% MLBS/1.00 BPP) or high (≥90% MLBS/ ≥0.95 BPP). Values lower than 70% MLBS and 0.95 BPP were considered to be of low support and not indicated in phylogenetic trees.

Differences in SSU, ITS, LSU, *RPB2*, and *TEF1* sequences amongst the various species of *Splanchospora* were calculated using MEGA v.7.0. Table 2 illustrates the variation in intraspecific conserved sites amongst the *Splanchospora* species.

**Table 2.** Comparison of intraspecific conserved sites amongst *Splanchospora* species.

	ITS1 (1–143)								ITS2 (303–452)																	
site number	49	53	79	82	132	135	136	141	340	341	342	343	344	362	363	379	387	404	414	419	422	444				
*S. ampullacea* (5 strains)	C	C	C	A	A	A	C	G	C	G	C	C	T	C	T	C	C	C	T	A	G	T				
*S. fulviconidia* (6 strains^|^)	G	**T**	C	A	C/-	**T**	T	**A**	**T**	**A**	-^†^	-^†^	-^†^	T	T	C	C	A	A	G	**G**	**A**				
*S. microspora* (2 strains)	G	C	C	A	-	A	T	G	C	G	C	**T**	T	T	C	T	C	C	T	A	G	T				
*S. oblongiconidia* (3 strains)	G	C	C	**G**	A	A	C	G	C	G	C	C	T	C	C	C	C	C	T	A	A	T				
*S. pseudomicrospora* (7 strains^|^)	G	C	C	A	A	A	C	G	C	G	C	C	T	T/C	C/T	C	C	C	T	A	G	T				
*Splanchospora* sp. (1 strain)	C	C	**T**	A	A	A	C	G	C	G	C	C	T	C	T	C	T	C	T	A	G	T				
	SSU (1–1,346)											LSU (1–1,320)														
site number	490–807	884	885	887	935	969	977	978	979	980	988	56	58	60	99	371	373	410	526	624	655	662	1026	1248	1267	1285
*S. ampullacea*	318 sites^‡^	T^‡^	G^‡^	G^‡^	T^‡^	T^‡^	G^‡^	-^‡^	G^‡^	A^‡^	C^‡^	A	T	T	T	G	G	C	T	G	C	G	C^§^	C^§^	G^§^	C^§^
*S. fulviconidia*	-	**G**	**A**	**A**	-^†^	**C**	**A**	**C**	**T**	**C**	**A**	**T**	**A**	**A**	C	**A**	**A**	**T**	**A**	**A**	**T**	G	**T**	**T**	**A**	**T**
*S. microspora*	-	T	G	G	T	T	G	-	G	A	C	A	T	T	C	G	G	C	T	G	C	**C**	C	C	G	C
*S. oblongiconidia*	-	T	G	G	T	T	G	-	G	A	C	A	T	T	T	G	G	C	T	G	C	G	C	C	G	C
*S. pseudomicrospora*	318 sites	T	G	G	T	T	G	-	G	A	C	A	T	T	T	G	G	C	T	G	C	G	C	C	G	C
*Splanchospora* sp.	n/a^¶^	n/a^¶^	n/a^¶^	n/a^¶^	n/a^¶^	n/a^¶^	n/a^¶^	n/a^¶^	n/a^¶^	n/a^¶^	n/a^¶^	A	T	T	T	G	G	C	T	G	C	G	n/a^¶^	n/a^¶^	n/a^¶^	n/a^¶^
	*RPB2* exon (1–1,065)																									
site number	18	45	81	84	87	90	120	127	132	139	147	150	153	189	192	198	219	291	294	297	303					
*S. ampullacea*	T	C	T	C	C	C	T	C	A	T	G	C	T	G	C	A	C	G	**G**	G	C					
*S. fulviconidia*	**C**	**T**	C	C	**T**	C	**C**	**T**	G	**C**	G	C	T	**C**	C	A	**A**	G	A	G	**T**					
*S. microspora*	T	C	T	C	C	C	T	C	A	T	G	C	C	G	C	A	C	G	A	G	C					
*S. oblongiconidia*	T	C	C	**T**	C	**T**	T	C	A	T	**A**	C	C	G	**T**	A	C	**A**	A	G	C					
*S. pseudomicrospora*	T	C	C	C	C	C	**G**	C	G	T	G	**A**	C	G	C	**G**	C	G	A	G	C					
*Splanchospora* sp.	T	C	T	C	C	C	T	C	A	T	G	C	C	G	C	A	C	G	A	**A**	C					
	*RPB2* exon (1–1,065)																									
site number	306	309	312	333	336	360	366	372	378	396	399	456	465	480	483	492	501	504	510	522	537					
*S. ampullacea*	C	T	C	T	G	C	C	C	G	T	C	**C**	T	T	A	C	C	C	T	T	**G**					
*S. fulviconidia*	C	**C**	**T**	C	**A**	T	T	**T**	A	**C**	**T**	T	T	T	A	C	**T**	C	**C**	T	A					
*S. microspora*	C	T	C	T	G	C	C	C	G	T	C	T	T	T	A	C	C	C	T	T	A					
*S. oblongiconidia*	**T**	T	C	C	G	T	T	C	A	T	C	**G**	C	T	**C**	**G**	C	**T**	T	**C**	A					
*S. pseudomicrospora*	C	T	C	C	G	C	C	C	G	T	C	T	C	**C**	A	C	C	C	T	T	A					
*Splanchospora* sp.	C	T	C	T	G	C	C	C	G	T	C	T	T	T	A	C	C	C	T	T	A					
	*RPB2* exon (1–1,065)																									
site number	570	579	609	618	624	636	642	657	666	667	675	684	687	708	711	714	726	729	**737**	741	765					
*S. ampullacea*	C	G	T	T	A	T	G	C	C	C	T	C	T	T	C	C	A	G	C	G	C					
*S. fulviconidia*	**A**	G	A	T	A	C	A	**T**	T	T	T	**T**	T	**C**	C	C	G	A	**G**	G	C					
*S. microspora*	C	G	T	T	A	C	G	C	C	C	T	C	T	T	C	C	A	G	C	G	C					
*S. oblongiconidia*	C	**A**	A	**A**	A	C	A	C	T	C	T	C	C	T	C	T	G	A	C	G	**C**					
*S. pseudomicrospora*	C	G	A	T	**G**	C	A	C	T	T	T	C	C	T	**T**	T	G	A	C	**A**	C					
*Splanchospora* sp.	C	G	T	T	A	T	G	C	C	C	**C**	C	T	T	C	C	A	G	C	G	**T**					
	*RPB2* exon (1–1,065)																									
site number	768	780	783	789	795	831	861	873	918	**928**	930	936	942	963	**973**	975	1020	1026	1041	1047	1050	1056				
*S. ampullacea*	G	C	T	A	A	T	G	T	G	A	T	T	C	T	C	T	A	T	T	C	C	C				
*S. fulviconidia*	**T**	C	T	A	A	T	A	**C**	G	A	C	T	C	T	C	T	G	**C**	**C**	**T**	T	T				
*S. microspora*	G	C	**C**	A	A	T	G	T	G	G	C	T	C	T	C	T	A	T	T	C	C	T				
*S. oblongiconidia*	G	**T**	T	G	A	T	G	T	**C**	G	C	**C**	**T**	**A**	C	**C**	G	T	T	C	T	T				
*S. pseudomicrospora*	G	C	T	G	**G**	**C**	A	T	G	G	C	T	C	T	**A**	T	G	T	T	C	T	T				
*Splanchospora* sp.	G	C	T	A	A	T	G	T	G	G	T	T	C	T	C	T	A	T	T	C	C	C				
	*TEF1* intron 1 (1–270)																									
site number	5	17	28	33	45	46	114	117	128	157	163	164	168	169	173	174	182									
*S. ampullacea*	T	T	C	A	-	-	T	C	G	T	A	T	A	T	G	T	A									
*S. fulviconidia*	T	A	**G**	**G**	**T**	**T**	G	C	**T**	T	**C**	**C**	A	T	**A**	**A**	**G**									
*S. microspora*	**C**	T	C	A	-	-	G	C	G	**C**	A	T	A	T	G	T	A									
*S. oblongiconidia*	T	A	C	A	-	-	G	C	G	T	A	T	C	**C**	G	T	A									
*S. pseudomicrospora*	T	T	C	A	-	-	G/T	C	G	T	A	T	A	T	G	T	A									
*Splanchospora* sp.	T	T	C	A	-	-	G	**T**	G	T	A	T	C	T	G	T	A									
	*TEF1* intron 1 (1–270)																	exon 1 (271–409)								
site number	183	186	201–221	222	227	228	229	231	242	246	248	249	250	258	262	263	264	396								
*S. ampullacea*	T/C	A	-	A	T	T	G	C	**A**	C	C	A	A	A	**C**	C	T	T								
*S. fulviconidia*	T	A	**21 sites**	C	**C**	**A**	**T**	**A**	G	**G**	**A**	A	**G**	A	T	C	T	**C**								
*S. microspora*	T	**C**	-	A	T	T	G	C	G	C	C	A	A	A	T	C	T	T								
*S. oblongiconidia*	T	A	-	C	T	T	G	C	G	C	C	**C**	A	A	T	C	C	T								
*S. pseudomicrospora*	C	A	-	A	T	T	G	C	G	C	C	A	A	A	T	C	C	T								
*Splanchospora* sp.	T	A	-	A	T	T	G	C	G	C	C	A	A	**G**	T	**T**	C	T								
	*TEF1* intron 2 (410–459)														exon 2 (460–1,253)											
site number	412	417	418	422	423	424	425	426	427	431	437	438	439	446	467	518	635	638	641	659	677	704	752			
*S. ampullacea*	A	A	T	T	A	A	T	G	C	G/C	G	G	T	T	C	C	T	C	C	C	T	C	T			
*S. fulviconidia*	A	**C**	**C**	T	C	A	-	G	T	**A**	G	**T**	C	T	C	**T**	C/T	C	C	**T**	**C**	C	**C**			
*S. microspora*	A	A	T	-^†^	-^†^	-^†^	-	**C**	T	G	G	G	C	T	C	C	T	C	**T**	C	T	**G**	T			
*S. oblongiconidia*	**G**	A	T	T	C	A	T	G	C	G	G	G	C	**C**	**T**	C	C	**T**	C	C	T	C	T			
*S. pseudomicrospora*	A	A	T	T	**T**	A	T	G	C	G	G	G	T	T	C	C	T	C	C	C	T	C	T			
*Splanchospora* sp.	A	A	T	T	A	A	T	G	C	G	**A**	G	C	T	C	C	T	C	C	C	T	C	T			
	*TEF1* exon 2 (460–1,253)																									
site number	758	767	776	788	**798**	**799**	872	893	923	986	1058	1070	1073	**1086**	1097	1103	1106	1160	1178	1196	1211	1226	1235	1253		
*S. ampullacea*	C	T	T	C	A	C	T	G	G	T	G	G	G	C	C	C	T	C	G	C	T	**T**	G	T		
*S. fulviconidia*	C	T	**C**	T	A	C	C	C	**A**	**C**	G	**A**	G	**G**	C	C	T	T	**A**	**T**	T	C	G	**C**		
*S. microspora*	**T**	C	T	T	A	C	T	G	G	T	**A**	G	G	C	**T**	C	C	C	G	C	T	C	**A**	T		
*S. oblongiconidia*	C	C	T	C	**T**	C	C	C	G	T	G	G	G	C	C	C	C	T	G	C	T	C	G	T		
*S. pseudomicrospora*	C	T	T	C	A	**G**	T	G	G	T	G	G	G	C	C	**T**	C	T	G	C	**C**	C	G	T		
*Splanchospora* sp.	C	T	T	C	**G**	C	T	G	G	T	G	G	**A**	C	C	C	C	C	G	C	T	C	G	T		

A hyphen (‘-’) indicates a gap site. Nucleotide in bold and gap with ^†^ indicate that only a particular species has a different base or a gap from all other species. Site number in bold indicates substitution causing missense mutation. ‡ The SSU sequences of *S. ampullacea* were determined using only CBS 116578 and L20. § The region between LR5 and LR7 in LSU sequences of *S. ampullacea* was determined using only CBS 116578. | For the comparison of ITS sequences only, 9 strains each of *S. fulviconidia* and *S. pseudomicrospora* were used. ¶ SSU and the region between LR5 and LR7 in LSU of *Splanchospora* sp. (L181) were not sequenced.

## Results

### Molecular phylogenetic and barcode analyses

**Figure 1.**
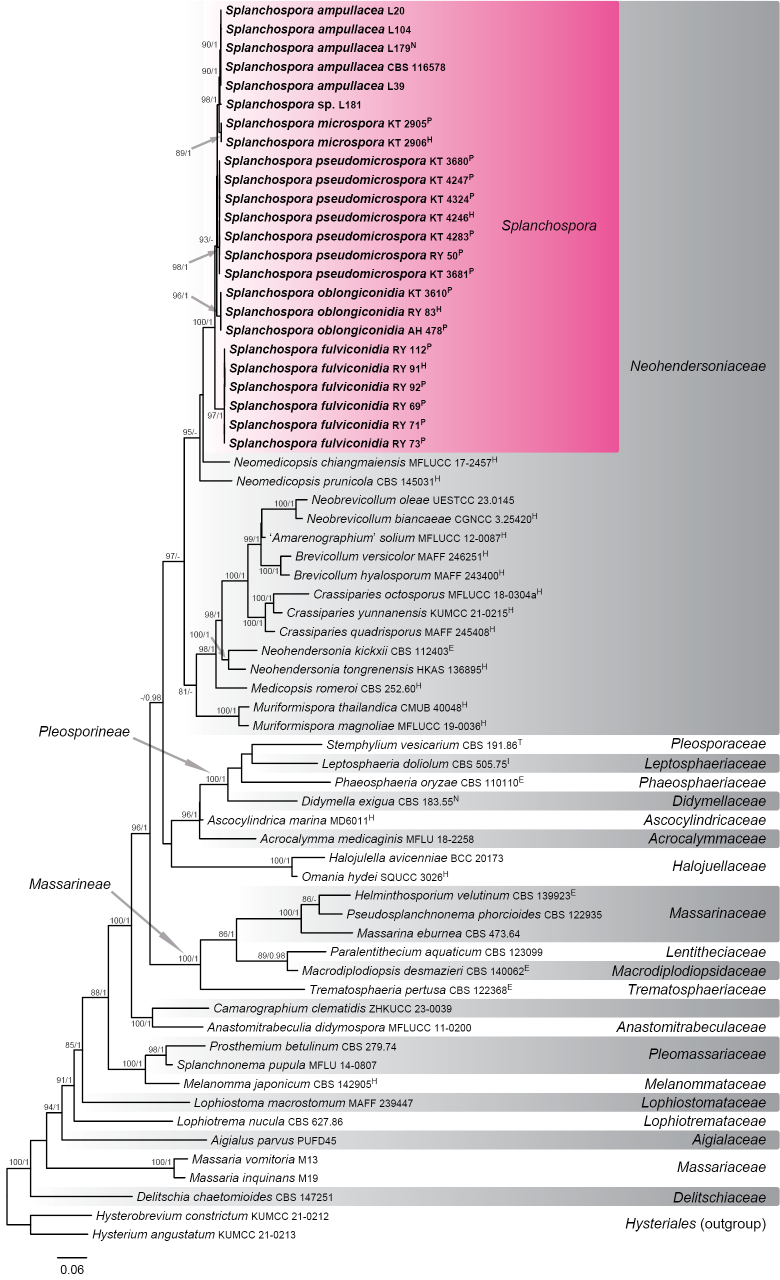
Maximum-likelihood (ML) tree of *Pleosporales* based on combined SSU, ITS, LSU, *RPB2*, and *TEF1* regions. ML bootstrap support (MLBS) values above 70% and Bayesian posterior probability (BPP) values above 0.95 are presented at the nodes as MLBS/BPP. A hyphen (‘-’) indicates values lower than 70% MLBS or 0.95 BPP. The newly obtained sequences are shown in bold. The scale bar represents nucleotide substitutions per site. Ex-epitype, ex-holotype, ex-isotype, ex-neotype, ex-paratype, and ex-type strains are indicated as E, H, I, N, P, and T, respectively.

In the first analysis, ML and Bayesian phylogenetic trees were generated using an aligned sequence dataset comprising SSU, ITS, LSU, *RPB2*, and *TEF1* sequences. The dataset and statistics resulting from the phylogenetic analyses (e.g. the number of characters and selected substitution models) are provided in Suppl. material 1: table S3. The ML tree with the highest log-likelihood (InL = −39,388.245) is shown in Fig. 1. The phylogram provided a higher confidence topology and values for familial classification than the individual gene trees did, with 20 families reconstructed in *Pleosporales*. The topology recovered by the Bayesian analysis was almost identical to that of the ML tree, except for the branching order between two *Neomedicopsis* species. Members of *Splanchospora* formed a monophyletic and fully supported clade (100% MLBS/1.0 BPP) within the family *Neohendersoniaceae*. *Splanchospora* was found to be closely related to the genus *Neomedicopsis*. However, their relationships were not fully supported, and *Neomedicopsis* appeared to be polyphyletic in the ML and Bayesian trees.

**Figure 2.**
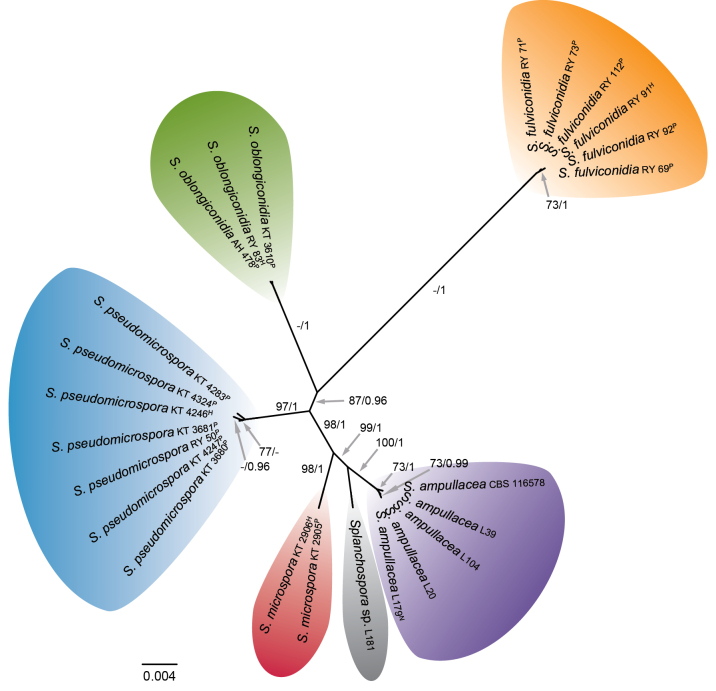
Maximum-likelihood (ML) tree of *Splanchospora* based on combined ITS, *RPB2* and *TEF1* regions. ML bootstrap support (MLBS) values above 70% and Bayesian posterior probability (BPP) values above 0.95 are presented at the nodes as MLBS/BPP. A hyphen (‘-’) indicates values lower than 70% MLBS or 0.95 BPP. The scale bar represents nucleotide substitutions per site. Ex-holotype, ex-neotype, and ex-paratype strains are indicated as H, N, and P, respectively.

In the second analysis, ML and Bayesian phylogenetic trees based on the ITS, *RPB2*, and *TEF1* sequences were generated using an aligned sequence dataset of members of *Splanchospora*. The statistics and models used for these datasets are listed in Suppl. material 1: table S3. The ML trees with the highest log-likelihoods (InL = −771.13881 in ITS, −1,919.8091 in *RPB2*, −2,353.4835 in *TEF1*, and −5,099.5852 in ITS-*RPB2*-*TEF1*) are shown in Fig. 2 and Suppl. material 1: fig. S1. The topology recovered by the Bayesian analyses was almost identical to that of the ML trees, except for the branching order amongst species in the *TEF1* tree. The combined tree revealed six distinct lineages, indicating the existence of six species. These were the type species *S. ampullacea* and the cryptic species (*Splanchospora* sp., L181), both from Europe, and four newly identified species from Japan (*Splanchospora fulviconidia***sp. nov**., *Splanchospora microspora***sp. nov**., *Splanchospora oblongiconidia***sp. nov**., and *Splanchospora pseudomicrospora***sp. nov**.). *Splanchospora ampullacea*, *S. microspora*, and *S. pseudomicrospora* formed a highly supported monophyletic clade (≥97% MLBS/1.0 BPP). *Splanchospora fulviconidia* and *S. oblongiconidia* had low MLBS values (<70% MLBS/1.0 BPP; Fig. 2), whereas the single-gene trees based on *RPB2* and *TEF1* formed fully supported clades (100% MLBS/1.0 BPP) of the two species (Suppl. material 1: fig. S1B, C) and the ITS tree formed a fully supported clade of *S. fulviconidia* and a highly supported clade of *S. oblongiconidia* (91% MLBS/1.0 BPP) (Suppl. material 1: fig. S1A).

Amongst the examined loci, interspecies variation was observed at 22 of 452 sites in ITS, 10 of 1,346 sites in SSU, 15 of 1,320 sites in LSU, 85 of 1,065 sites in *RPB2*, and 102 of 1,253 sites in *TEF1* (Table 2). Of the 5,436 sites in total, the numbers of sites with distinct bases or gaps in each species compared with those in the other species were as follows: *S. ampullacea*, 6; *S. fulviconidia*, 117; *S. microspora*, 17; *S. oblongiconidia*, 28; *S. pseudomicrospora*, 14; and *Splanchospora* sp., 11. All the SSU sequences of *S. ampullacea* (CBS 116578 and L20) and *S. pseudomicrospora* (7 strains) contained a 318 bp insertion.

### Taxonomy

#### Neohendersoniaceae

Taxon classificationFungiPleosporalesNeohendersoniaceae

Giraldo & Crous, Mycol. Progr. 16 (4): 343 (2017)

BC95C543-D81A-55C2-BE14-EF05EEACA462

##### Type genus.

*Neohendersonia*.

##### Description.

**Sexual morph**. Ascomata pseudoperithecial, scattered, immersed, erumpent at the ostiolar neck, globose to depressed globose or ampulliform, ostiolate. Ostiolar neck central to excentric. Ascomatal wall composed of brown cells. Pseudoparaphyses septate, branched and anastomosed. Asci bitunicate, fissitunicate, cylindrical to clavate or pyriform, pedicellate. Ascospores broadly ellipsoidal to clavate-ellipsoid or broadly fusiform, 1-septate to multi-septate or muriform, hyaline or pigmented, smooth or faintly roughened.

**Asexual morph**. Conidiomata pycnidial, solitary or gregarious, immersed, globose to depressed globose or ampulliform. Ostiolar neck central. Conidiomatal wall composed of brown cells. Paraphyses absent or present, septate, branched. Conidiophores absent. Conidiogenous cells discrete, determinate or indeterminate, annellidic or phialidic, cylindrical, lageniform, doliiform or ampulliform, hyaline, smooth. Conidia globose to ellipsoid, obovoid, cylindrical, clavate or fusiform, septate or aseptate, hyaline or pigmented. Spermatia subglobose to ellipsoidal.

##### Notes.

*Neohendersoniaceae* accommodates eight genera, *Brevicollum*, *Crassiparies*, *Medicopsis*, *Muriformispora*, *Neobrevicollum*, *Neohendersonia*, *Neomedicopsis*, and *Splanchospora*. ‘*Amarenographium*’ *solium* is also a member of this family, but its generic placement remains uncertain (Tanaka et al. 2017). *Crassiparies* (Li et al. 2016; Tanaka et al. 2017), *Medicopsis* (Devadatha et al. 2020), *Muriformispora* (de Silva et al. 2022; Wu et al. 2024), *Neomedicopsis* (Hyde et al. 2018; Crous et al. 2019), and *Splanchospora* (this study) are known to have both sexual and asexual morphs. On the other hand, *Brevicollum* (Tanaka et al. 2017) and *Neobrevicollum* (Lu et al. 2024) are known only from their sexual morphs, whereas *Neohendersonia* (Giraldo et al. 2017) and ‘*Amarenographium*’ *solium* (Hodhod et al. 2012) are known only from their asexual morphs. In this study, we show that the presence of paraphyses in the conidiomata of *Splanchospora* expands the morphological concept of the asexual stage in *Neohendersoniaceae*.

#### Splanchospora

Taxon classificationFungiPleosporalesNeohendersoniaceae

Lar.N. Vassiljeva, Nizshie Rasteniya, Griby i Mokhoobraznye Dalnego Vostoka Rossii 4: 237 (1998)

BF552B1D-3B7E-50B6-937F-1D066A97827C

##### Type species.

*Splanchospora ampullacea*

##### Description.

**Sexual morph**. Ascomata pseudoperithecial, scattered, immersed, erumpent at the ostiolar neck, subglobose to depressed globose or ampulliform, ostiolate. Ostiolar neck central, developed or less developed. Ascomatal wall composed of brown cells. Pseudoparaphyses septate, branched and anastomosed. Asci bitunicate, clavate, short-stalked, with 8, rarely 2–7 ascospores. Ascospores clavate-ellipsoid, 1-septate, with a submedian septum, strongly constricted at the septum, thick-walled, pale to dark brown, smooth or faintly roughened, with an entire sheath.

**Asexual morph**. Conidiomata pycnidial, scattered, immersed, subglobose to depressed globose or ampulliform. Ostiolar neck central. Conidiomatal wall composed of brown cells. Paraphyses septate, branched. Conidiophores absent. Conidiogenous cells annellidic, cylindrical to ampulliform. Conidia globose to ellipsoid, aseptate, hyaline or yellowish brown, smooth, thick-walled.

##### Notes.

*Splanchospora* formed a monophyletic clade that was distinctly separated from any genera in the family *Neohendersoniaceae* (Fig. 1). *Splanchospora* is similar to the genera with sexual morphs in having immersed ascomata with a central ostiolar neck, except for *Brevicollum*, which has an excentric to ostiolar neck (Tanaka et al. 2017). However, *Splanchospora* differs from *Crassiparies*, *Medicopsis*, *Muriformispora*, *Neobrevicollum*, and *Neomedicopsis* in having clavate-ellipsoid, thick-walled ascospores strongly constricted at the submedian septum. Amongst the members of *Neohendersoniaceae* with asexual morphs, *Neohendersonia* and *Neomedicopsis* are more similar to *Splanchospora* in having annellidic conidiogenous cells (those of *Neomedicopsis* were described as ‘proliferating percurrently’ and illustrated in fig. 34B in Crous et al. 2019) and thick-walled conidia. Notably, *Neomedicopsis* shares long conidiogenous cells and aseptate conidia with *Splanchospora* and is sister to the latter genus. However, *Splanchospora* is distinguished from *Neohendersonia* and *Neomedicopsis* by the presence of paraphyses in the conidiomata.

#### Splanchospora ampullacea

Taxon classificationFungiPleosporalesNeohendersoniaceae

(Pers.) Lar.N. Vassiljeva, Nizshie Rasteniya, Griby i Mokhoobraznye Dalnego Vostoka Rossii 4: 238 (1998)

6EF5D60F-95CB-568F-8092-BD1A2F2006B1

Figs 4, 10 A, F

≡ Splanchnonema ampullaceum (Pers.) Shoemaker & P.M. LeClair, Canad. J. Bot. 53(15): 1570 (1975).
= Massaria curreyi Tul. & C. Tul. (as ‘*curreii*’), Selecta Fungorum Carpologia, Tomus Secundus. Xylariei - Valsei - Sphaeriei 2: 231 (1863).
≡ Massariella curreyi (Tul. & C. Tul.) Sacc., Syll. Fung. 1: 717 (1882).

##### Basionym.

*Sphaeria ampullacea* Pers., Synopsis methodica fungorum: 41 (1801).

**Figure 3.**
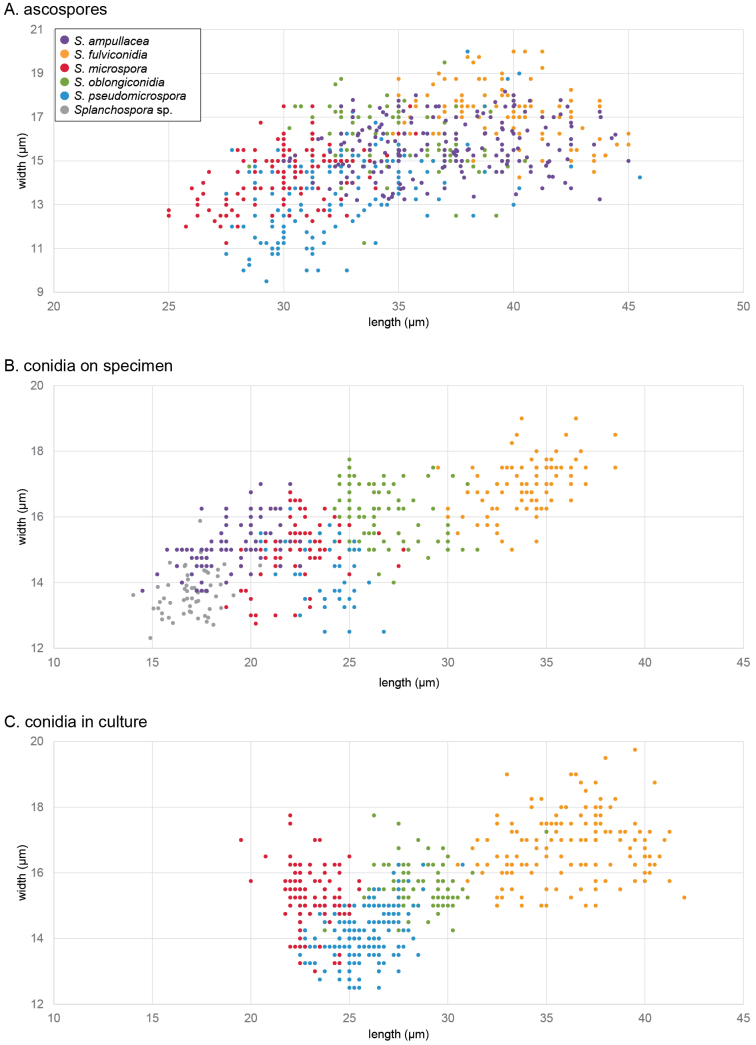
Scatter plot comparisons of spore size amongst *Splanchospora* species. **A** ascospores; **B** conidia on specimen; **C** conidia in culture. The vertical and horizontal axes indicate width and length, respectively. The purple, orange, red, green, blue, and gray circles represent spore sizes of *S. ampullacea*, *S. fulviconidia*, *S. microspora*, *S. oblongiconidia*, *S. pseudomicrospora*, and *Splanchospora* sp., respectively.

**Figure 4.**
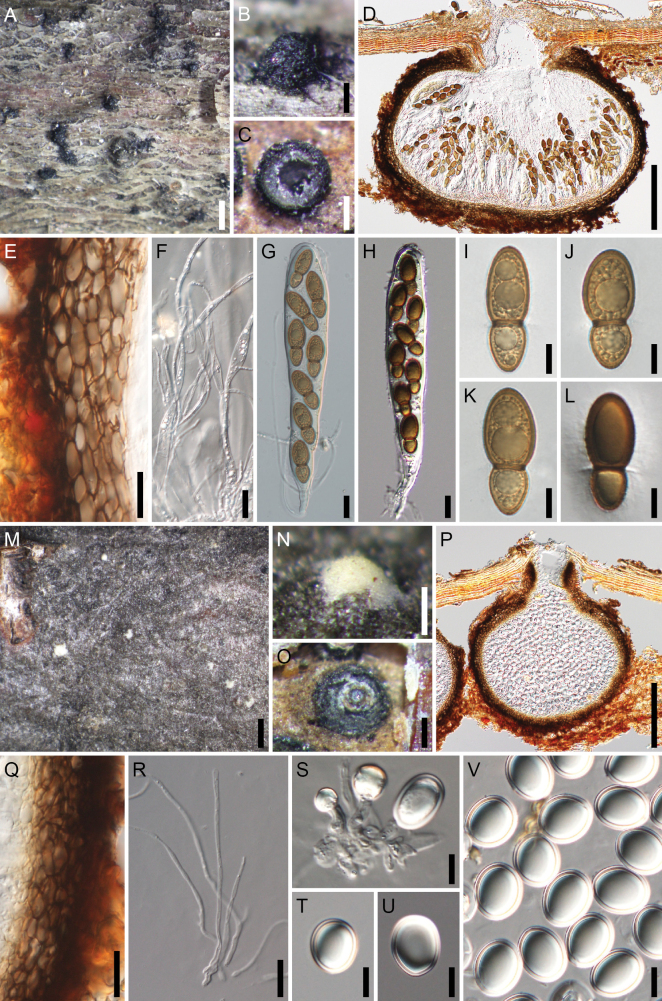
*Splanchospora ampullacea*. **A–L** Sexual morph. **M–V** Asexual morph. **A** Ascomata in face view. **B** Slimy mass of ascospores exuded though the ostiole of ascoma. **C** Ascoma in lateral section. **D** Ascoma in longitudinal section. **E** Ascomatal wall at side. **F** Pseudoparaphyses. **G, H** Asci. **I–L** Ascospores. **M** Conidiomata in face view. **N** Slimy mass of conidia exuded though the ostiole of conidioma. **O** Conidioma under bark. **P** Conidioma in longitudinal section. **Q** Conidiomatal wall at side. **R** Paraphyses. **S** Conidiogenous cells and immature conidia. **T–V** Conidia. Specimen: L179 (**A–V**). Scale bars: 1 mm (**A, M**); 200 μm (**B–D, N–P**); 20 μm (**E–H, Q, R**); 10 μm (**I–L, S–V**).

**Figure 5.**
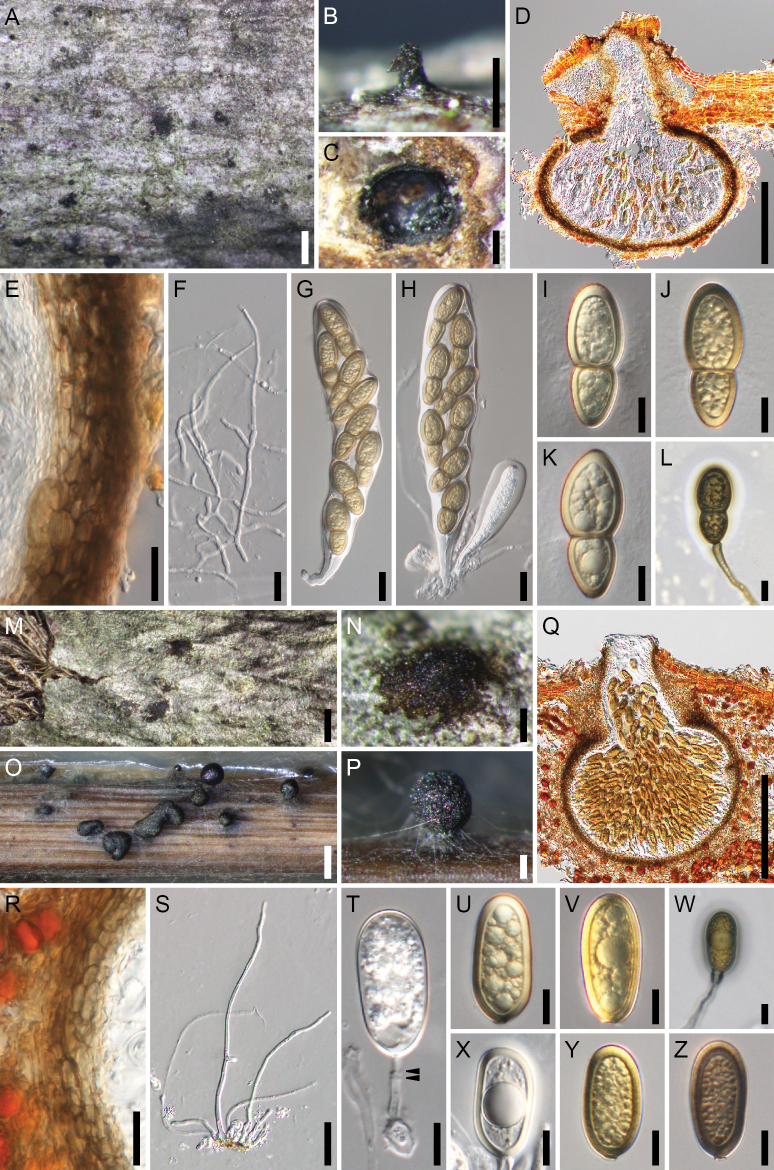
*Splanchospora fulviconidia*. **A–L** Sexual morph. **M–Z** Asexual morph. **A** Ascomata in face view. **B** Slimy mass of ascospores exuded though the ostiole of ascoma. **C** Ascoma in lateral section. **D** Ascoma in longitudinal section. **E** Ascomatal wall at side. **F** Pseudoparaphyses. **G, H** Asci. **I–K** Ascospores. **L** Germinating ascospore. **M, O** Conidiomata in face view (**M** on natural substrate, **O** on a rice straw in culture). **N, P** Slimy mass of conidia exuded though the ostiole of conidiomata (**N** on natural substrate, **P** on a rice straw in culture). **Q** Conidioma in longitudinal section. **R** Conidiomatal wall at side. **S** Paraphyses. **T** Conidiogenous cell and immature conidium (arrowheads indicate annellations). **U, V, X–Z** Conidia (**U, V** from specimens; **X–Z** from culture; **X** immature; **Z** senescent). **W** Germinating conidium. Specimens: RY 73 (**A–E, G, H, J, L**); RY 91 (**F, K**); RY 69 (**I**); RY 71 (**M, N, Q, R, V, W**); RY 112 (**U**). Strains: culture RY 92 (**O, P, S, X**); culture RY 71 (**T, Y**); culture RY 91 (**Z**). Scale bars: 1 mm (**A, M, O**); 200 μm (**B–D, N, P, Q**); 20 μm (**E–H, R, S**); 10 μm (**I–L, T–Z**).

**Figure 6.**
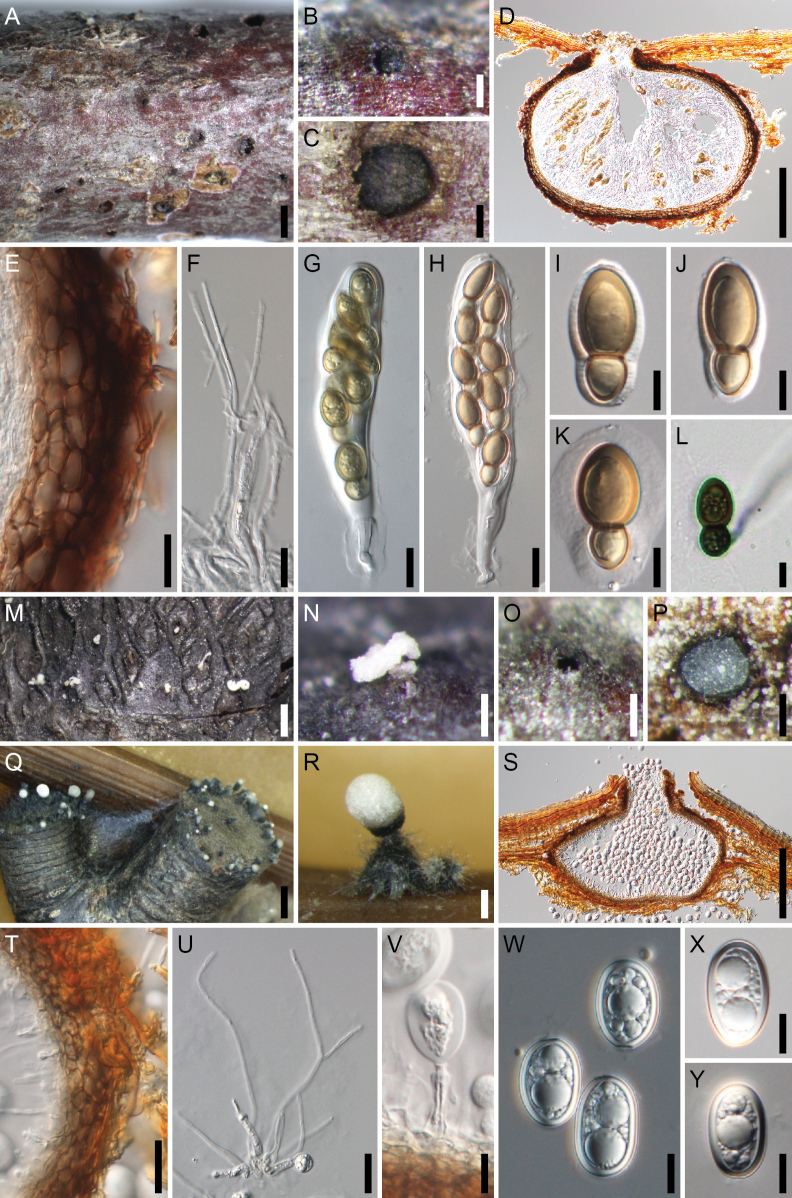
*Splanchospora microspora*. **A–L** Sexual morph. **M–Y** Asexual morph. **A** Ascomata in face view. **B** Ostiole in face view. **C** Ascoma in lateral section. **D** Ascoma in longitudinal section. **E** Ascomatal wall at side. **F** Pseudoparaphyses. **G, H** Asci. **I–K** Ascospores. **L** Germinating ascospore. **M, Q** Conidiomata in face view (**M** on natural substrate, **Q** on a beech twig in culture). **N, R** Slimy mass of conidia exuded though the ostiole of conidiomata (**N** on natural substrate, **R** on a rice straw in culture). **O** Ostiole in face view. **P** Conidioma in lateral section. **S** Conidioma in longitudinal section. **T** Conidiomatal wall at side. **U** Paraphyses. **V** Conidiogenous cell and immature conidium. **W–Y** Conidia (**W** from specimen; **X, Y** from culture). Specimens: KT 2906 (**A–L**); KT 2905 (**M–P, S, T, V, W**). Strains: culture KT 2905 (**Q, R, U, X**); culture KT 2906 (**Y**). Scale bars: 1 mm (**A, M, Q**); 200 μm (**B–D, N–P, R, S**); 20 μm (**E–H, T, U**); 10 μm (**I–L, V–Y**).

**Figure 7.**
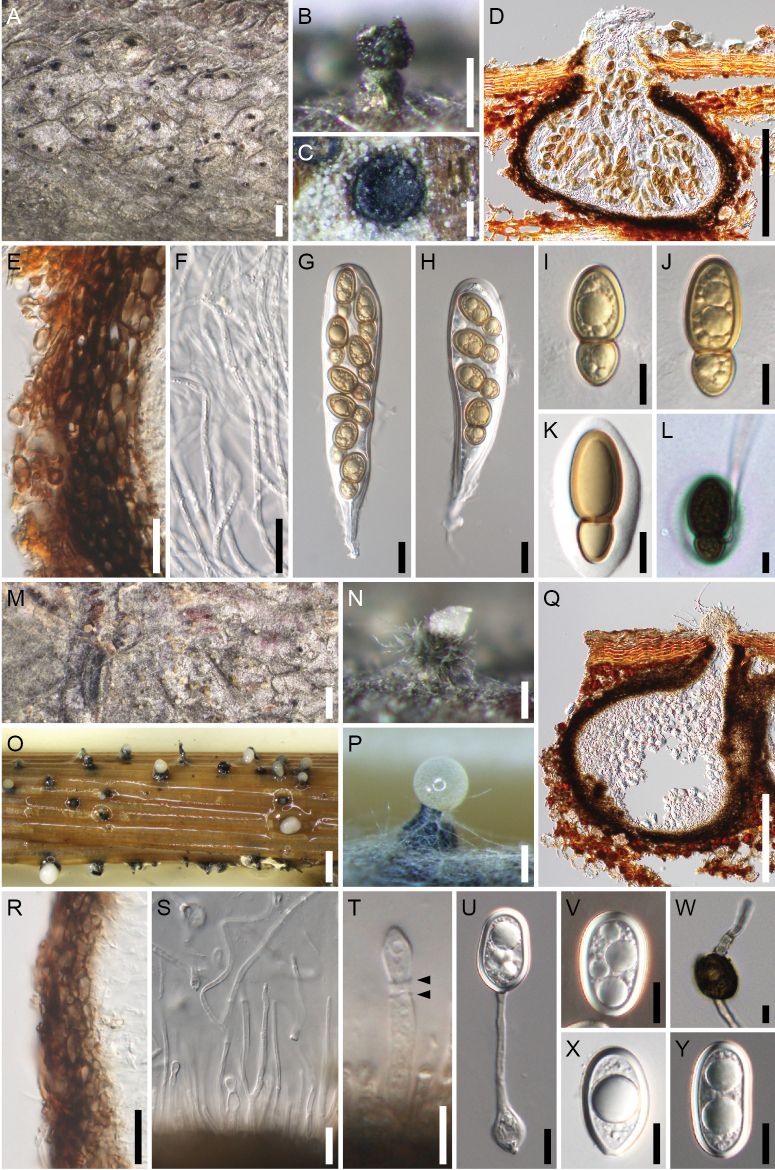
*Splanchospora oblongiconidia*. **A–L** Sexual morph. **M–Y** Asexual morph. **A** Ascomata in face view. **B** Slimy mass of ascospores exuded though the ostiole of ascoma. **C** Ascoma in lateral section. **D** Ascoma in longitudinal section. **E** Ascomatal wall at side. **F** Pseudoparaphyses. **G, H** Asci. **I–K** Ascospores. **L** Germinating ascospore. **M, O** Conidiomata in face view (**M** on natural substrate, **O** on a rice straw in culture). **N, P** Slimy mass of conidia exuded though the ostiole of conidiomata (**N** on natural substrate, **P** on a beech twig in culture). **Q** Conidioma in longitudinal section. **R** Conidiomatal wall at side. **S** Paraphyses. **T** Conidiogenous cell (arrowheads indicate annellations). **U** Conidiogenous cell and conidium. **V, X, Y** Conidia (**V** from specimen; **X, Y** from culture). **W** Germinating conidium. Specimens: RY 83 (**A–D, F–J, M, N, Q**); AH 478 (**E, K, L**); KT 3610 (**V, W**). Strains: culture RY 83 (**O, S–U, Y**); culture AH 478 (**P, R, X**). Scale bars: 1 mm (**A, M, O**); 200 μm (**B–D, N, P, Q**); 20 μm (**E–H, R, S**); 10 μm (**I–L, T–Y**).

**Figure 8.**
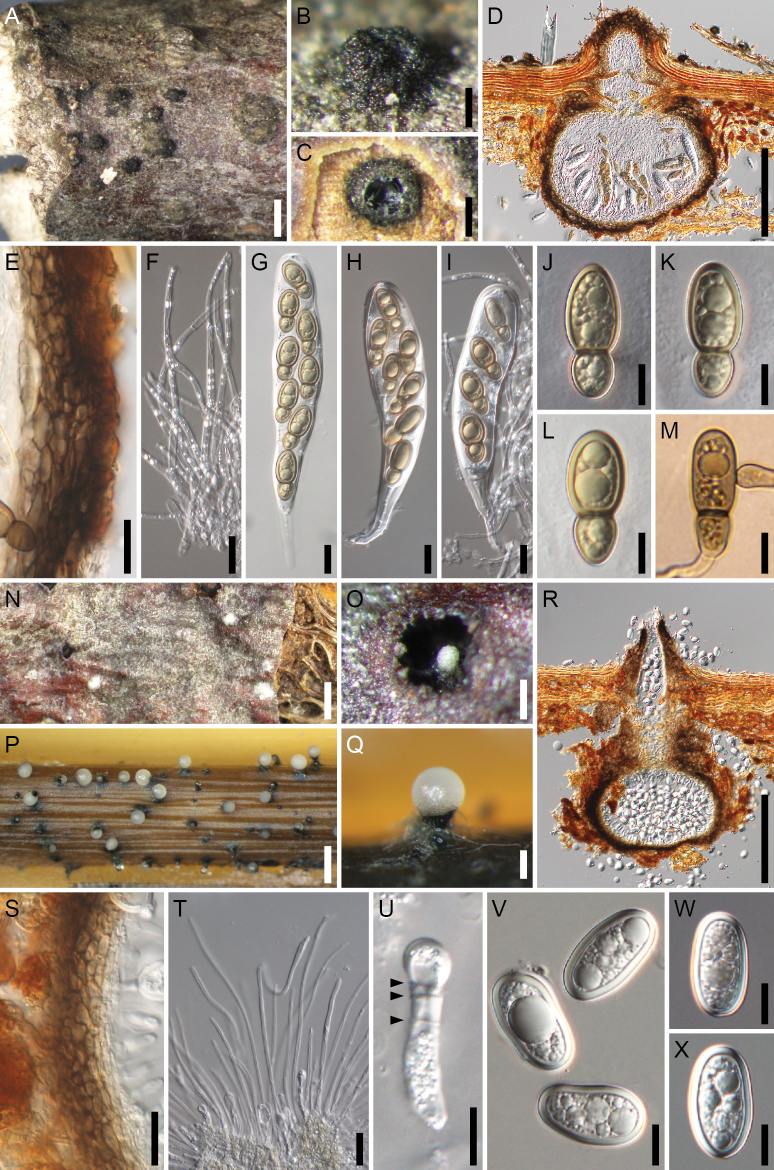
*Splanchospora pseudomicrospora*. **A–M** Sexual morph. **N–X** Asexual morph. **A** Ascomata in face view. **B** Slimy mass of ascospores exuded though the ostiole of ascoma. **C** Ascoma in lateral section. **D** Ascoma in longitudinal section. **E** Ascomatal wall at side. **F** Pseudoparaphyses. **G–I** Asci. **J–L** Ascospores. **M** Germinating ascospore. **N, P** Conidiomata in face view (**N** on natural substrate, **P** on a rice straw in culture). **O, Q** Slimy mass of conidia exuded though the ostiole of conidiomata (**O** on natural substrate, **Q** on a beech twig in culture). **R** Conidioma in longitudinal section. **S** Conidiomatal wall at side. **T** Paraphyses. **U** Conidiogenous cell and immature conidium (arrowheads indicate annellations). **V–X** Conidia (**V** from specimen; **W, X** from culture). Specimens: RY 50 (**A–I, L**); KT 3680 (**J**); KT 4246 (**K, M**); KT 4247 (**N, O, R, S**); KT 4324 (**V**). Strains: culture KT 4247 (**P, Q, T**); culture KT 4324 (**U, X**); culture RY 50 (**W**). Scale bars: 1 mm (**A, N, P**); 200 μm (**B–D, O, Q, R**); 20 μm (**E–I, S, T**); 10 μm (**J–M, U–X**).

**Figure 9.**
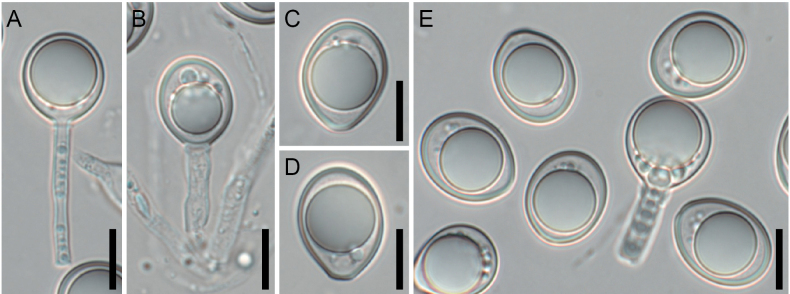
*Splanchospora* sp. **A, B** Conidiogenous cells and conidia. **C–E** Conidia. Specimen: L181 (**A–E**). Scale bars: 10 μm (**A–E**).

**Figure 10.**
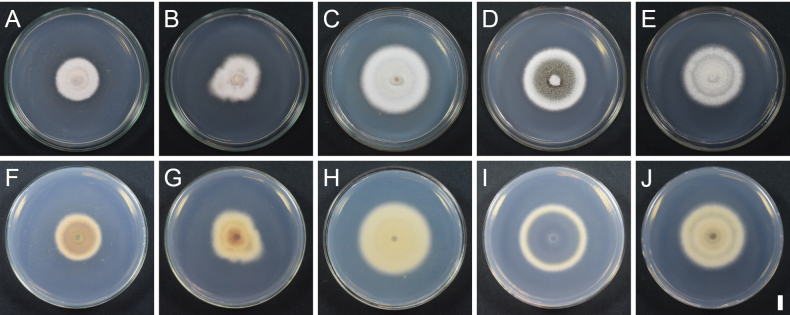
Colony characters of *Splanchospora* species on PDA at 20 °C in the dark after 2 weeks. **A–E** Surface; **F–J** Reverse. Species: *S. ampullacea* (CBS 116578) (**A, F**); *S. fulviconidia* (culture RY 91 = MAFF 248153) (**B, G**); *S. microspora* (culture KT 2906 = MAFF 248150) (**C, H**); *S. oblongiconidia* (culture RY 83 = MAFF 248152) (**D, I**); *S. pseudomicrospora* (culture KT 4246 = MAFF 248151) (**E, J**). Scale bar: 1 cm.

##### Type.

AUSTRIA • Burgenland, Hornstein, Lindenallee, *T. cordata* Mill., 4 Nov 2017, H. Voglmayr & I. Greilhuber L179 (**neotype**WU-MYC 0057753 here designated, MBT 10029611, **isoneotype**HHUF 30704, ex-neotype CBS 154683 = culture L179).

##### Description.

**Sexual morph**. Ascomata pseudoperithecial, scattered, immersed, erumpent at the ostiolar neck, subglobose to depressed globose, 480–770 μm high, 530–800 μm diam, ostiolate. Ostiolar neck central, 80–120 μm long, 110–180 μm wide. Ascomatal wall (24–)35–45 μm thick at the sides, composed of 5–6 layers of polygonal, 10–21 × 5–8(–9) μm, brown cells. Pseudoparaphyses 2.5–4 μm wide, septate, branched and anastomosed. Asci bitunicate, clavate, 181–230(–245) × 30–39 μm (mean ± SD = 212.6 ± 13.3 × 34.4 ± 2.2 μm, n = 33), short-stalked (30–70 μm long, mean ± SD = 46.1 ± 11.1 μm, n = 33), 8-spored, rarely 4-, 6- or 7-spored due to meiotic failure or stunted spores, with uni- to bi-seriate ascospores. Ascospores clavate-ellipsoid, 30–45 × 13–18 μm (mean ± SD = 37.0 ± 3.4 × 15.6 ± 1.2 μm, n = 197), l/w = 1.9–3.1(–3.3) (mean ± SD = 2.4 ± 0.3, n = 197), 1-septate, with a submedian septum [(0.55–)0.58–0.67(–0.70), mean ± SD = 0.63 ± 0.03, n = 100], strongly constricted at the septum, brown to dark brown, smooth to faintly roughened, with an entire sheath.

**Asexual morph**. Conidiomata pycnidial, scattered, immersed, ampulliform, 310–560 μm high, 240–450 μm diam, ostiolate. Ostiolar neck central, 80–140 μm long, 100–220 μm wide. Conidiomatal wall 20–47 μm thick, composed of 4–6 layers of polygonal, 7.5–16 × 4.5–8 μm, brown cells. Paraphyses 2.5–3.5 μm wide, septate, branched. Conidiophores absent. Conidiogenous cells annellidic, cylindrical, (12–)15–23(–29) × 3–7.5(–11) μm (mean ± SD = 19.8 ± 5.1 × 5.8 ± 2.0 μm, n = 13). Conidia globose to subglobose, 14.5–22.5 × 14–17 μm (mean ± SD = 18.8 ± 1.7 × 15.1 ± 0.7 μm, n = 102), l/w = 1.1–1.4(–1.6) (mean ± SD = 1.2 ± 0.1, n = 102), aseptate, hyaline, smooth, with thick spore wall (1–1.5 μm).

Colonies on PDA (after 2 weeks) attaining a diam of 2.7–3.2 cm, white; reverse isabelline; no pigment produced.

##### Distribution.

Asia [Georgia (Nakhutsrishvili 1986), the Russian Far East (Vasilyeva 1998)]; Europe [Austria (this study), Belgium, Czech Republic, Denmark (Munk 1957), Finland, France, Germany, Hungary, Italy, Lithuania (Treigienė 1999), Luxembourg, Netherlands, Norway (Nordén et al. 2018), Poland (Mułenko et al. 2008), Portugal, Russia (Mel’nik 2011), Spain, Sweden (Eriksson 2014; this study), Switzerland (Tonolo 1956), UK (Pirozynski and Morgan-Jones 1968; Barr 1982; this study), Ukraine]; North America [Canada, USA (Barr 1982)]. Records of countries without references are based on specimens recorded in Cybertruffle (http://www.cybertruffle.org.uk/eng/), GBIF Secretariat (2023), or MyCoPortal (https://www.mycoportal.org/portal/index.php); true species identities of collections outside of Europe require additional studies.

##### Additional specimens examined.

AUSTRIA • Wien, 3. Bez., Botanical Garden of the University of Vienna (HBV), *Tilia platyphyllos* Scop., 4 Apr 2006, H. Voglmayr, L20 (WU-MYC 0057750 = HHUF 30703; CBS 122932 = culture L20); Wien, 21. Bez., Donauinsel near Nordbrücke, *Tilia cf. cordata*, 5 May 2013, W. Jaklitsch, L104 (WU-MYC 0057752, culture L104). UK • London, Richmond, Kew Gardens, *Tilia* sp., 11 Nov 2008, H. Voglmayr, L39 (WU-MYC 0057751, CBS 124477 = culture L39).

##### Additional strain examined.

SWEDEN • Uppland, Dalby parish, Ekbacken, *Tilia cordata*, 9 Mar 1991, K. & L. Holm, CBS 116578 = UPSC 3376.

##### Notes.

For further synonyms, please refer to Barr (1982). The location of the type specimen of *Sphaeria ampullacea* is unknown, and the original description of the species did not provide information on the ascospores (Persoon 1801). The only extant collection of *Sphaeria ampullacea* in the Persoon herbarium (L0112460) cannot represent the type, because this collection was evidently collected after its description (Persoon 1801), as brought forward in a detailed note by R. A. Shoemaker attached to this specimen (see https://bioportal.naturalis.nl/nl/multimedia/L0112460). To stabilize the species concept, we therefore here designate collection WU-MYC 0057753, for which detailed morphological data as well as sequences are available, as the neotype for *Sphaeria ampullacea* Persoon. Shoemaker and LeClair (1975) designated the neotype specimen of *Massaria curreyi*, which is a heterotypic synonym of *Sphaeria ampullacea*, and examined its ascospores. Although the ascospores from our specimens (30–45 × 13–18 μm) are broader than those from the neotype of *Massaria curreyi* (36–42 × 13–14.5 μm; Shoemaker and LeClair 1975), the values partially overlap. In our specimens, pycnidia with 1-celled conidia are closely associated with the species. Although the strains examined in this study were sterile in culture, we recognised the pycnidia on the natural specimens as being asexual morphs on the basis of their similarity to those of other *Splanchospora* spp.

#### Splanchospora fulviconidia

Taxon classificationFungiPleosporalesNeohendersoniaceae

R. Yoshioka, Voglmayr & Kaz. Tanaka sp. nov.

CB97A67B-BB2D-5EB4-BCE9-526B4EFA716C

860946

Figs 5, 10 B, G

##### Etymology.

From the Latin *fulvi*, meaning tawny, in reference to the tawny conidia.

##### Diagnosis.

*Splanchospora fulviconidia* can be easily distinguished from other species by its large-sized, yellowish-brown conidia.

##### Type.

JAPAN • Ibaraki: Tsukuba, Kannondai, Forest Research and Management Organization, Arboretum 2, dead twigs of *Tilia japonica* (Miq.) Simonk., 27 Mar 2024, R. Yoshioka, Y. Kudo & K. Tanaka, RY 91 (**holotype**HHUF 30708, ex-type MAFF 248153 = culture RY 91).

##### Description.

**Sexual morph**. Ascomata pseudoperithecial, scattered, immersed, erumpent at the ostiolar neck, subglobose to depressed globose or ampulliform, 560–570 μm high, 500–600 μm diam, ostiolate. Ostiolar neck central, 90–120 μm long, 80–110 μm wide. Ascomatal wall 34–39 μm thick at the sides, composed of 4–5 layers of polygonal, 6–14.5(–17) × 4–7 μm, brown cells. Pseudoparaphyses 2.5–3.5 μm wide, septate, branched and anastomosed. Asci bitunicate, clavate, 162–212(–296) × 27.5–41(–44) μm (mean ± SD = 196.3 ± 23.0 × 34.1 ± 3.3 μm, n = 62), short-stalked (25–63 μm long, mean ± SD = 40.1 ± 11.5 μm, n = 11), 8-spored, rarely 2- or 6-spored due to meiotic failure or stunted spores, with bi- to tri-seriate ascospores. Ascospores clavate-ellipsoid to fusoid, 34.5–45 × 14–20 μm (mean ± SD = 39.4 ± 2.4 × 17.1 ± 1.2 μm, n = 128), l/w = 1.9–3.0 (mean ± SD = 2.3 ± 0.3, n = 128), 1-septate, with a submedian septum [0.54–0.62(–0.68), mean ± SD = 0.59 ± 0.02, n = 128), strongly constricted at the septum, pale brown to brown, smooth, with an entire sheath.

**Asexual morph**. Conidiomata pycnidial, scattered, immersed, subglobose to depressed globose or ampulliform, 460–490 μm high, 350–460 μm diam, ostiolate. Ostiolar neck central, 150–170 μm long, 100–110 μm wide. Conidiomatal wall 19–26 μm thick, composed of 4–5 layers of polygonal, 5–11(–12.5) × 2.5–5 μm, brown cells. Paraphyses 2.5–4 μm wide, septate, branched. Conidiophores absent. Conidiogenous cells annellidic, cylindrical to ampulliform, 11–17.5(–20) × 3–5 μm (mean ± SD = 15.6 ± 2.3 × 4.2 ± 0.6 μm, n = 11). Conidia ellipsoid to irregularly ellipsoid, 29.5–37(–38.5) × 15–19 μm (mean ± SD = 33.9 ± 1.9 × 17.0 ± 0.8 μm, n = 90), l/w = (1.7–)1.8–2.2(–2.3) (mean ± SD = 2.0 ± 0.1, n = 90), aseptate, pale to yellowish brown, becoming dark brown during senescence, smooth, with thick spore wall (2–3 μm).

Colonies on PDA (after 2 weeks) attaining a diam of 3.2–4 cm, white; reverse sienna; no pigment produced. In culture same coelomycetous asexual morph formed. Conidia 30.5–42 × 15–20 μm (mean ± SD = 36.2 ± 2.7 × 16.8 ± 1.0 μm, n = 150), l/w = (1.7–)1.8–2.5(–2.8) (mean ± SD = 2.2 ± 0.2, n = 150).

##### Distribution.

Japan.

##### Additional specimens examined.

JAPAN • Aomori: Aomori, Komagome, Fukazawa, Tsukimino Forest Park, dead twigs of *T. japonica* or *T. maximowicziana* Shiraz., 14 Oct 2023, R. Yoshioka, Y. Kudo & K. Tanaka, RY 69 (**paratype**HHUF 30705, culture RY 69); ibid., RY 71 (**paratype**HHUF 30706, culture RY 71); ibid., RY 73 (**paratype**HHUF 30707, culture RY 73); ibid., dead twigs of *T. japonica* or *T. maximowicziana*, 9 Sep 2024, R. Yoshioka & Y. Kudo, RY 152 (**paratype**HHUF 30711, culture RY 152); ibid., RY 153 (**paratype**HHUF 30712, culture RY 153); ibid., RY 161 (**paratype**HHUF 30713, culture RY 161); Ibaraki: Tsukuba, Kannondai, Forest Research and Management Organization, Arboretum 2, dead twigs of *T. japonica*, 27 Mar 2024, R. Yoshioka, Y. Kudo & K. Tanaka, RY 92 (**paratype**HHUF 30709, culture RY 92); Tsukuba, Matsunosato, Forest Research and Management Organization, Arboretum 1, dead twigs of *T. kiusiana*, 27 Mar 2024, R. Yoshioka, Y. Kudo & K. Tanaka, RY 112 (**paratype**HHUF 30710, culture RY 112).

##### Notes.

*Splanchospora fulviconidia* differs from all other *Splanchospora* species in having yellowish-brown conidia. Moreover, the conidia [29.5–37(–38.5) × 15–19 μm] are the largest amongst the six *Splanchospora* species examined. The position of the primary septum in the ascospores (mean 0.59) is more central than that of the other *Splanchospora* species (mean ≥0.63).

#### Splanchospora microspora

Taxon classificationFungiPleosporalesNeohendersoniaceae

R. Yoshioka, Voglmayr & Kaz. Tanaka sp. nov.

F77A4A81-9641-5A26-923E-1E2079EA6F03

860947

Figs 6, 10 C, H

##### Etymology.

From the Greek *micro*, meaning small, referring to the small ascospores and conidia of this species relative to those of other *Splanchospora* species in Japan.

**Diagnosis**. *Splanchospora microspora* can be distinguished from *S. ampullacea* in having a thicker conidial wall and being native to Japan.

**Type**. JAPAN • Aomori: Aomori, Komagome, Fukazawa, Tsukimino Forest Park, dead twigs of *Tilia* sp., 19 Sep 2011, K. Tanaka, KT 2906 (**holotype**HHUF 30715, ex-type MAFF 248150 = culture KT 2906).

**Description**

**Sexual morph**. Ascomata pseudoperithecial, scattered, immersed, depressed globose, 320–570 μm high, 410–700 μm diam, ostiolate. Ostiolar neck central, 60–100 μm long, 80–200 μm wide. Ascomatal wall 23–30 μm thick at the sides, composed of 4–5 layers of polygonal, 6.5–16(–29) × 3–6.5(–8.5) μm, brown cells. Pseudoparaphyses 2–3 μm wide, septate, branched and anastomosed. Asci bitunicate, clavate, (118–)127–179 × 27.5–35 μm (mean ± SD = 155.4 ± 20.1 × 30.8 ± 2.4 μm, n = 14), short-stalked (23–50 μm long, mean ± SD = 37.5 ± 7.9 μm, n = 14), 8-spored, rarely 4-, or 6-spored due to meiotic failure, with uni- to tri-seriate ascospores. Ascospores clavate-ellipsoid, 25–36 × (11–)12–17.5 μm (mean ± SD = 30.0 ± 2.1 × 14.3 ± 1.3 μm, n = 137), l/w = (1.7–)1.8–2.5(–2.6) (mean ± SD = 2.1 ± 0.2, n = 137), 1-septate, with a submedian septum (0.59–0.71, mean ± SD = 0.65 ± 0.02, n = 137), strongly constricted at the septum, pale brown to brown, smooth, with an entire sheath.

**Asexual morph**. Conidiomata pycnidial, scattered, immersed, depressed globose, 280–500 μm high, 380–520 μm diam, ostiolate. Ostiolar neck central, 65–110 μm long, 90–160 μm wide. Conidiomatal wall 27–37 μm thick, composed of 4–6 layers of polygonal, 6–11.5 × 3–6(–7) μm, brown cells. Paraphyses 2–3 μm wide, septate, branched. Conidiophores absent. Conidiogenous cells annellidic, cylindrical to ampulliform, 6.5–19(–23) × 3–5(–6.5) μm (mean ± SD = 11.7 ± 3.8 × 4.7 ± 0.5 μm, n = 42). Conidia subglobose to ellipsoid, 19–25(–28) × 13–17 μm (mean ± SD = 22.3 ± 1.7 × 15.0 ± 0.9 μm, n = 80), l/w = 1.3–1.8(–1.9) (mean ± SD = 1.5 ± 0.1, n = 80), aseptate, hyaline, smooth, with thick spore wall (2.5–3 μm).

Colonies on PDA (after 2 weeks) attaining a diam of 4.6–4.8 cm, white; reverse buff; no pigment produced. In culture same coelomycetous asexual morph formed. Conidia 19–25.5 × 13–18 μm (mean ± SD = 23.1 ± 1.0 × 15.3 ± 0.9 μm, n = 112), l/w = (1.1–)1.3–1.7(–1.8) (mean ± SD = 1.5 ± 0.1, n = 112).

##### Distribution.

Japan.

##### Additional specimen examined.

JAPAN • Aomori: Aomori, Komagome, Fukazawa, Tsukimino Forest Park, dead twigs of *Tilia* sp., 19 Sep 2011, K. Tanaka, KT 2905 (**paratype**HHUF 30714, culture KT 2905).

##### Notes.

*Splanchospora microspora* is phylogenetically close to *S. ampullacea* and *Splanchospora* sp. from Europe (Fig. 2, Suppl. material 1: fig. S1B). However, its conidial length [19–25(–28) μm, mean 22.3 μm] is somewhat longer than that of *S. ampullacea* (14.5–22.5 μm, mean 18.8 μm) and *Splanchospora* sp. (14–20.5 μm, mean 17.0 μm). Moreover, the conidial wall of *S. microspora* (2.5–3 μm) is thicker than that of *S. ampullacea* (1–1.5 μm) and *Splanchospora* sp. (0.9–1.1 μm).

#### Splanchospora oblongiconidia

Taxon classificationFungiPleosporalesNeohendersoniaceae

R. Yoshioka, Voglmayr & Kaz. Tanaka sp. nov.

E4AE44F2-6CDB-5238-A9CD-113155A2E922

860948

Figs 7, 10 D, I

##### Etymology.

Meaning oblong, referring to the longest conidia of *Splanchospora* species with hyaline ones.

##### Diagnosis.

*Splanchospora oblongiconidia* can be distinguished from *S. microspora* and *S. pseudomicrospora* by its longer conidia.

##### Type.

JAPAN • Aomori: Aomori, Katta, Heiwa Park, dead twigs of *T. maximowicziana*, 22 Oct 2022, Y. Kudo, RY 83 (**holotype**HHUF 30718, ex-type MAFF 248152 = culture RY 83).

##### Description.

**Sexual morph**. Ascomata pseudoperithecial, scattered, immersed, erumpent at the ostiolar neck, subglobose to depressed globose, 310–560 μm high, 320–750 μm diam, ostiolate. Ostiolar neck central, 90–160 μm long, 175–200 μm wide. Ascomatal wall (19–)29–55(–60) μm thick at the sides, composed of 4–6 layers of polygonal, 7–19 × 3–10 μm, brown cells. Pseudoparaphyses 2.5–4 μm wide, septate, branched and anastomosed. Asci bitunicate, clavate, (125–)139–193 × 29.5–38(–42) μm (mean ± SD = 163.2 ± 16.9 × 34.3 ± 3.3 μm, n = 46), short-stalked (24–53 μm long, mean ± SD = 37.6 ± 7.5 μm, n = 35), 8-spored, rarely 2- to 7-spored due to meiotic failure or stunted spores, with uni- to tri-seriate ascospores. Ascospores clavate-ellipsoid, (28.5–)30–40.5 × (11.5–)14–18(–19.5) μm (mean ± SD = 34.7 ± 2.5 × 15.8 ± 1.3 μm, n = 111), l/w = 1.7–2.7(–3.1) (mean ± SD = 2.2 ± 0.3, n = 111), 1-septate, with a submedian septum [(0.57–)0.60–0.71, mean ± SD = 0.65 ± 0.03, n = 111], strongly constricted at the septum, pale brown to brown, smooth, with an entire sheath.

**Asexual morph**. Conidiomata pycnidial, scattered, immersed, erumpent at the ostiolar neck, subglobose, 470–490 μm high, 400–450 μm diam, ostiolate. Ostiolar neck central, 140–165 μm long, 100–125 μm wide. Conidiomatal wall 23–52 μm thick, composed of 4–5 layers of polygonal, 7.5–15(–17) × 3.5–8 μm, brown cells. Paraphyses 3–3.5 μm wide, septate, branched. Conidiophores absent. Conidiogenous cells annellidic, cylindrical to ampulliform, (14.5–)18.5–31 × 3–6 μm (mean ± SD = 23.1 ± 4.7 × 4.2 ± 0.9 μm, n = 10). Conidia ellipsoid to irregularly ellipsoid, 24–31.5 × 14–18 μm (mean ± SD = 26.6 ± 1.8 × 16.1 ± 0.9 μm, n = 100), l/w = 1.4–2.1 (mean ± SD = 1.7 ± 0.2, n = 100), aseptate, hyaline, smooth, with thick spore wall (2–3 μm).

Colonies on PDA (after 2 weeks) attaining a diam of 4–4.5 cm, olivaceous grey at central part, white at margin; reverse olivaceous grey at central part, buff at margin; no pigment produced. In culture same coelomycetous asexual morph formed. Conidia (24–)26–31(–35) × 14–18 μm (mean ± SD = 28.3 ± 1.4 × 15.5 ± 0.7 μm, n = 110), l/w = 1.5–2.1 (mean ± SD = 1.8 ± 0.1, n = 110).

##### Distribution.

Japan.

##### Additional specimens examined.

JAPAN • Hokkaido: Sapporo, Chuo-ku, Mt. Maruyama, dead twigs of *Ostrya japonica*, 17 Oct 2015, K. Tanaka, KT 3610 (**paratype**HHUF 30717, culture KT 3610); Nagano: Ueda, Sugadaira Highland, University of Tsukuba, Mountain Science Center, Sugadaira Research Station, dead twigs of *Carpinus cordata*, 2 Jul 2016, A. Hashimoto, AH 478 (**paratype**HHUF 30716, culture AH 478).

##### Notes.

The conidia of *S. oblongiconidia* (24–31.5 × 14–18 μm) are longer than those of *S. microspora* [19–25(–28) × 13–17 μm] and *S. pseudomicrospora* [20.5–25.5(–27) × 12.5–16 μm] and shorter those of than *S. fulviconidia* [29.5–37(–38.5) × 15–19 μm]. In addition to *Tilia maximowicziana*, *S. oblongiconidia* was found to be associated with *Carpinus cordata* and *Ostrya japonica*, indicating that *Splanchospora* spp. may have some latitude in host selection, although their main host is linden trees.

#### Splanchospora pseudomicrospora

Taxon classificationFungiPleosporalesNeohendersoniaceae

R. Yoshioka, Voglmayr & Kaz. Tanaka sp. nov.

DF4E4780-B9B9-5052-B478-6205CE7B279E

860949

Figs 8, 10 E, J

##### Etymology.

From the Greek *pseudo*, meaning spurious, in reference to morphological similarity to *Splanchospora microspora*.

##### Diagnosis.

It can be distinguished from *S. microspora* by its slightly elongated, slender conidia and variable-sized ascospores.

##### Type.

JAPAN • Aomori: Tsugaru, Kizukuritateoka, Bense Marsh, dead twigs of *T. japonica*, 28 Aug 2021, K. Tanaka, KT 4246 (**holotype**HHUF 30721, ex-type MAFF 248151 = culture KT 4246).

##### Description.

**Sexual morph**. Ascomata pseudoperithecial, scattered, immersed, erumpent at the ostiolar neck, subglobose to depressed globose or ampulliform, 420–720 μm high, 410–560 μm diam, ostiolate. Ostiolar neck central, 170–200 μm long, 170–210 μm wide. Ascomatal wall (20–)30–39(–51) μm thick at the sides, composed of 4–5 layers of polygonal, 7–18(–22) × 3–7.5(–11) μm, brown cells. Pseudoparaphyses 2.5–4 μm wide, septate, branched and anastomosed. Asci bitunicate, clavate, 146–193(–212) × 27.5–36 μm (mean ± SD = 172.3 ± 16.5 × 31.5 ± 2.2 μm, n = 23), short-stalked (27–62 μm long, mean ± SD = 41.5 ± 9.7 μm, n = 17), 8-spored, rarely 2- to 7-spored due to meiotic failure or stunted spores, with uni- to bi-seriate ascospores. Ascospores clavate-ellipsoid, 27.5–37(–45.5) × 9.5–16(–20) μm (mean ± SD = 32.4 ± 3.2 × 13.7 ± 1.8 μm, n = 176), l/w = (1.8–)1.9–2.9(–3.3) (mean ± SD = 2.4 ± 0.3, n = 176), 1-septate, with a submedian septum (0.57–0.71, mean ± SD = 0.64 ± 0.02, n = 176), strongly constricted at the septum, pale brown to brown, smooth, with an entire sheath.

**Asexual morph**. Conidiomata pycnidial, scattered, immersed, erumpent at the ostiolar neck, ampulliform, 270–400 μm high, 500–550 μm diam, ostiolate. Ostiolar neck central, 210–350 μm long, 190–220 μm wide. Conidiomatal wall 23–30(–34) μm thick, composed of 4–5 layers of polygonal, 5.5–12(–15) × 2.5–6 μm, brown cells. Paraphyses 2.5–3.5 μm wide, septate, branched. Conidiophores absent. Conidiogenous cells annellidic, cylindrical to ampulliform, 12–22(–38) × (3–)5–8(–11) μm (mean ± SD = 18.2 ± 5.4 × 6.9 ± 1.9 μm, n = 30). Conidia ellipsoid to irregularly ellipsoid, 20.5–25.5(–27) × 12.5–16 μm (mean ± SD = 23.6 ± 1.5 × 14.5 ± 0.9 μm, n = 50), l/w = 1.3–1.9(–2.1) (mean ± SD = 1.6 ± 0.2, n = 50), aseptate, hyaline, smooth, with thick spore wall (2–3 μm).

Colonies on PDA (after 2 weeks) attaining a diam of 4.3–4.5 cm, white; reverse olivaceous grey, buff; no pigment produced. In culture same coelomycetous asexual morph formed. Conidia 22.5–29(–31) × 12.5–16 μm (mean ± SD = 25.4 ± 1.5 × 14.3 ± 0.8 μm, n = 230), l/w = (1.5–)1.6–2.0(–2.1) (mean ± SD = 1.8 ± 0.1, n = 230).

##### Distribution.

Japan.

##### Additional specimens examined.

JAPAN • Iwate: Hachimantai, Hirakasa, Iwate-san SA, dead twigs of *T. maximowicziana* or *T. platyphyllos*, 13 Aug 2016, KT 3680 (**paratype**HHUF 30719, culture KT 3680); ibid., KT 3681 (**paratype**HHUF 30720, culture KT 3681); ibid., dead twigs of *T. maximowicziana*, 2 Jan 2022, K. Tanaka, KT 4283 (**paratype**HHUF 30723, culture KT 4283); Aomori: Tsugaru, Kizukuritateoka, Bense Marsh, dead twigs of *T. japonica*, 28 Aug 2021, K. Tanaka, KT 4247 (**paratype**HHUF 30722, culture KT4247); Hirosaki, Shimoshirogane, Hirosaki Castle Botanical Garden, dead twigs of *T. japonica*, 5 Jun 2022, K. Tanaka, KT 4324 (**paratype**HHUF 30724, culture KT 4324); Aomori, Komagome, Fukazawa, Tsukimino Forest Park, dead twigs of *T. japonica* or *T. maximowicziana*, 19 Sep 2024, R. Yoshioka & Y. Kudo, RY 154 (**paratype**HHUF 30726, culture RY 154); ibid., RY 158 (**paratype**HHUF 30727, culture RY 158); Hokkaido: Sapporo, Shiroishi-ku, Nango-dori Avenue, Bansei Park, dead twigs of *T. japonica*, 18 Jun 2023, R. Yoshioka, RY 50 (**paratype**HHUF 30725, culture RY 50).

##### Notes.

Although *S. pseudomicrospora* is similar to *S. microspora*, the conidia of the former (mean 23.6 × 14.5 μm in the specimen and 25.4 × 14.3 μm in culture) are slightly longer and narrower than those of the latter (mean 22.3 × 15.0 μm in the specimen and 23.1 × 15.3 μm in culture) (Fig. 3B, C). These two species differ in various sequences: namely, at four sites with one gap in the ITS (99.1% homology = 448/452), at 27 sites with a single amino acid substitution in *RPB2* (97.5% = 1,038/1,065), and at 24 sites with four gaps and a single amino acid substitution in *TEF1* (98.0% = 1,206/1,230).

The ascospores size [27.5–37(–45.5) × 9.5–16(–20) μm (n = 176)] of *S. pseudomicrospora* is highly variable. This is probably due to occasional meiotic failure or stunted spores, resulting in enlargement of the remaining ascospores in the ascus. The size of ascospores from 8-spored asci are 27.5–37 × 11–15.5 μm (n = 54), whereas those from 4-spored asci are 38–40 × 17.5–20 μm (n = 4). This variability makes the identification of *Splanchospora* species on the basis of the ascospores alone more difficult.

#### Splanchospora

Taxon classificationFungiPleosporalesNeohendersoniaceae

sp.

ADC2BF09-AD45-5B61-9000-B7B205E14BD5

Fig. 9

##### Description.

**Sexual morph**. Not observed.

**Asexual morph**. Conidiogenous cells annellidic, cylindrical, 13–24 × 3–4 μm (mean ± SD = 17.0 ± 4.4 × 3.6 ± 0.3 μm, n = 4). Conidia globose to subglobose, 14–20.5 × 12.5–16 μm (mean ± SD = 17.0 ± 1.2 × 13.7 ± 0.6 μm, n = 56), l/w = 1.0–1.4 (mean ± SD = 1.2 ± 0.1, n = 56), aseptate, hyaline, smooth, with a spore wall thickness of 0.9–1.1 μm.

##### Specimen examined.

AUSTRIA • Wien, 3. Bez., Botanical Garden of the University of Vienna (HBV), *T. cordata*, 31 Jan 2018, H. Voglmayr, L181 (WU-MYC 0057754, culture L181).

##### Notes.

*Splanchospora* sp. is most closely related to *S. ampullacea* (Fig. 2, Suppl. material 1: fig. S1A, B). Sequence differences exist between these two species: namely, at two sites in the ITS (99.6% homology = 450/452), eight sites with a single amino acid substitution in *RPB2* (99.2% = 1,057/1,065), and 14 sites with a single amino acid substitution in *TEF1* (98.9% = 1,216/1,230). The conidia of the *Splanchospora* sp. (mean 17.0 × 13.7 μm) are slightly smaller than those of *S. ampullacea* (mean 18.8 × 15.1 μm). However, the only specimen (L181) of this fungus lacks sexual morph and was collected in the same place as a specimen of *S. ampullacea* (L20). For now, we have postponed the description of this fungus as a new species, pending the availability of additional materials with sexual morphs to avoid confusion owing to premature decision.

## Discussion

In this study, we have clarified the asexual morphs and phylogenetic position of *Splanchospora* for the first time. We provided a revised generic concept of *Splanchospora* on the basis of both its sexual and asexual characteristics and demonstrated the taxonomic validity of this genus in *Neohendersoniaceae*. Additionally, we identified four new species and discovered one undescribed species in this genus.

*Splanchospora ampullacea* was previously classified as *Splanchnonema* on the basis of the generic characteristics of having clavate, asymmetrically septate ascospores (Shoemaker and LeClair 1975). The genus *Splanchnonema* was established in 1829 with *Splanchnonema pustulatum* (current name: *Splanchnonema foedans*) as the type species and the asexual morph was revealed by Minter and Cannon (2013). In the asexual stage, these two genera share aseptate, thick-walled conidia and paraphyses (the latter in *Splanchnonema* were described as ‘paraphysis-like hyphae’ in Minter and Cannon 2013). They differ, however, in that the conidia of *Splanchospora* are globose to ellipsoid, while those of *Splanchnonema* are cylindrical to narrowly elongate. In the sexual stage, *Splanchospora* has 1-septate, thick-walled ascospores, whereas *Splanchnonema* has 2(rarely 3)-septate ascospores with an acute base (Zhang et al. 2012; Minter and Cannon 2013; Senn-Irlet et al. 2021).

*Splanchnonema* is known to be a polyphyletic group. To advance its taxonomic revision, information from the asexual morph is likely to be key for phylogenetic inference. For example, species whose asexual morphology differs from that of *Splanchnonema* sensu stricto have been excluded from this genus when considered together with molecular phylogenetic data: they are *Splanchnonema platani* (Ces.) M.E. Barr [current name: *Macrodiplodiopsis desmazieri* (Mont.) Petr.] with pycnidia producing mostly 3-septate, pigmented conidia (Crous et al. 2015, *Macrodiplodiopsidaceae*), *Splanchnonema phorcioides* (I. Miyake) P. Leroy, L. Gauthier & M.E. Barr, [current name: *Pseudosplanchnonema phorcioides* (I. Miyake) S. Konta, Camporesi & K.D. Hyde] with phoma-like asexual morph (Chethana et al. 2015, *Massarinaceae*), and *Splanchnonema kalakadense* Subram. & Sekar and *Splanchnonema quercicola* M.E. Barr (both are currently treated as *Helminthosporium*) with tretic, distoseptate conidia borne on erect conidiophores (Subramanian and Sekar 1987; Barr 1993; Voglmayr and Jaklitsch 2017, *Massarinaceae*). *Splanchnonema pupula* (Fr.) Kuntze, which produces acervuli with muriform conidia bearing lateral appendages (Tonolo 1956; Voglmayr and Jaklitsch 2008), should not be retained within *Splanchnonema* but should instead be placed in a separate genus, as previously proposed under the name *Stigmatomassaria* (nom. inval., Munk 1953). Further culture studies of other *Splanchnonema* species and the observation of their asexual morphs are expected to provide important clues to their phylogenetic placement. In addition, obtaining sequence data for the type species of *Splanchnonema* and clarifying its phylogenetic position remain essential tasks for the future.

In the genus *Splanchospora*, we identified the type species and one undescribed species from Europe, and described four new species from Japan. Although the ascospore sizes amongst these species overlapped except for the differentiation between *S. fulviconidia* and *S. microspora* (Fig. 3A), their conidial sizes (Fig. 3B, C), shapes, colours, wall thicknesses, and homology in DNA sequences were distinct. All six *Splanchospora* species were associated with dead twigs of linden trees. Our findings further support the host preference of *Splanchospora* for linden while also noting their occasional occurrences on *Fagales* trees, such as *Carpinus betulus* (Cybertruffle http://www.cybertruffle.org.uk/cgi-bin/robispec.pl?&colno=103169&glo=eng) and *Quercus macrocarpa* (The Kew Data Portal, https://records.data.kew.org/, Catalogue number K-M000458185) for *S. ampullacea*, and *C. cordata* and *O. japonica* for *S. oblongiconidia* (this study). *Splanchospora* species have multiple hosts and show overlap in their hosts. Our *S. ampullacea* specimens were associated with *T. cordata* and *T. platyphyllos* in Europe. The host, *T. japonica*, is shared by *S. fulviconidia* and *S. pseudomicrospora* in Japan. Interestingly, *S. fulviconidia* (RY 152) and *S. pseudomicrospora* (RY 154) were collected from the same linden individual at the same time. This indicates that although the two species co-exist in the same habitat, they are reproductively isolated.

*Splanchospora ampullacea* had been considered to be distributed throughout the mid-latitudes of the Northern Hemisphere and the only species in this genus. However, some specimens identified as *S. ampullacea* may be distinct species. The specimens in North America (Barr 1982) and the Russian Far East (Vasilyeva 1998) are not likely to be *S. ampullacea*. Based on specimens with identified host species, *S. ampullacea* in Europe is associated with *T. cordata* (Mel’nik 2011), *T. platyphyllos* (Nordén et al. 2018), *Tilia × europaea* K.Koch (as *Tilia × vulgaris*, The Kew Data Portal, Catalogue number K-M000084479), and *Tilia tomentosa* Moench (as *T. argentea*, Tonolo 1956), whereas the North American individuals are associated with *T. americana* and rarely *T. cordata* (MyCoPortal https://www.mycoportal.org/portal/index.php) and those from the Russian Far East are associated with *Tilia amurensis* Rupr. (Vasilyeva 1998). Considering the confusion caused by non-native linden trees, the fungal populations in these two regions may be considered distinct from those of the European species, reflecting differences in their original host species. As we identified four species of *Splanchospora* in Japan and a cryptic species of *S. ampullacea* in Europe, it is highly likely that multiple species also exist in North America and the Russian Far East.

The emphases on the ascospore morphology and host preference for species identification may be the reason why *S. ampullacea* has been the only species recognised for a long time. In this study, we found that the ascospore size was very similar amongst *Splanchospora* species (Fig. 3A). We also clarified that the preference for linden as the host is a common trait in this entire genus rather than a specific trait of *S. ampullacea*. Our results reveal that asexual characters, especially conidial features, combined with molecular data, provide valuable markers for species identification.

## Conclusion

By providing new morphological, ecological, and molecular data, this study enhances our understanding of both the sexual and asexual morphs of *Splanchospora* and clarifies their interspecific relationships. On the basis of phylogenetic analyses and comparative morphological studies, we re-enforce the generic circumscription of *Splanchospora* and classify this genus in the family *Neohendersoniaceae* (*Pleosporales*, *Dothideomycetes*). Furthermore, we recognise six species within this genus. Members of *Splanchospora* exist on dead twigs of *Tilia* spp. and co-exist on the same host species within their native region. Conidial morphology and secondary DNA barcodes, such as *TEF1* and *RPB2*, are useful for identifying species in this genus and will reveal unknown species diversity.

## Supplementary Material

XML Treatment for Neohendersoniaceae

XML Treatment for Splanchospora

XML Treatment for Splanchospora ampullacea

XML Treatment for Splanchospora fulviconidia

XML Treatment for Splanchospora microspora

XML Treatment for Splanchospora oblongiconidia

XML Treatment for Splanchospora pseudomicrospora

XML Treatment for Splanchospora

## Additional information

### Use of AI

No use of AI was reported.

### Adherence to national and international regulations

All the fungal strains used in this study have been legally obtained, respecting the Convention on Biological Diversity (Rio Convention).

### Author ORCIDs

Ryuichi Yoshioka https://orcid.org/0009-0001-3314-6343

Hermann Voglmayr https://orcid.org/0000-0001-7666-993X

Akira Hashimoto https://orcid.org/0000-0001-7834-3933

Kazuaki Tanaka https://orcid.org/0000-0002-7037-0774

### Data availability

The DNA sequences generated in this study are available on NCBI GenBank (https://www.ncbi.nlm.nih.gov). Sequence alignments are included in the Supplementary materials. All other data that support the findings of this study are available in the main text.

## Supplementary materials

Supplementary material 1 Additional figures and tables Data type pdf Explanation note **fig. S1**. Maximum-likelihood (ML) tree of *Splanchospora* based on each ITS (A), *RPB2* (B), and *TEF1* (C); **table SS1**. Specimens and isolates information of *Splanchospora* used in this study; **table SS2**. Primers used to amplify and sequence SSU, ITS, LSU, *RPB2*, and *TEF1*; **table S3**. Datasets used and statistics resulting from phylogenetic analyses in this study. https://binary.pensoft.net/file/1557641This dataset is made available under the Open Database License (http://opendatacommons.org/licenses/odbl/1.0/). The Open Database License (ODbL) is a license agreement intended to allow users to freely share, modify, and use this Dataset while maintaining this same freedom for others, provided that the original source and author(s) are credited. Ryuichi Yoshioka, Hermann Voglmayr, Akira Hashimoto, Misato Matsumura, Yoshihiro Kudo, Kazuaki Tanaka

Supplementary material 2 SSU alignment dataset used for the first analysis Data type fas Explanation note This dataset is composed of 24 strains of *Splanchospora* newly obtained in this study and 40 members of *Pleosporales* (including 15 members of *Neohendersoniaceae*) and 2 members of *Hysteriales* retrieved from GenBank. For missing SSU sequence data, see Table 1. https://binary.pensoft.net/file/1557642This dataset is made available under the Open Database License (http://opendatacommons.org/licenses/odbl/1.0/). The Open Database License (ODbL) is a license agreement intended to allow users to freely share, modify, and use this Dataset while maintaining this same freedom for others, provided that the original source and author(s) are credited. Ryuichi Yoshioka, Hermann Voglmayr, Akira Hashimoto, Misato Matsumura, Yoshihiro Kudo, Kazuaki Tanaka

Supplementary material 3 ITS alignment dataset used for the first analysis Data type fas Explanation note This dataset is composed of 24 strains of *Splanchospora* newly obtained in this study and 40 members of *Pleosporales* (including 15 members of *Neohendersoniaceae*) and 2 members of *Hysteriales* retrieved from GenBank. For missing ITS sequence data, see Table 1. https://binary.pensoft.net/file/1557643This dataset is made available under the Open Database License (http://opendatacommons.org/licenses/odbl/1.0/). The Open Database License (ODbL) is a license agreement intended to allow users to freely share, modify, and use this Dataset while maintaining this same freedom for others, provided that the original source and author(s) are credited. Ryuichi Yoshioka, Hermann Voglmayr, Akira Hashimoto, Misato Matsumura, Yoshihiro Kudo, Kazuaki Tanaka

Supplementary material 4 LSU alignment dataset used for the first analysis Data type fas Explanation note This dataset is composed of 24 strains of *Splanchospora* newly obtained in this study and 40 members of *Pleosporales* (including 15 members of *Neohendersoniaceae*) and 2 members of *Hysteriales* retrieved from GenBank. https://binary.pensoft.net/file/1557644This dataset is made available under the Open Database License (http://opendatacommons.org/licenses/odbl/1.0/). The Open Database License (ODbL) is a license agreement intended to allow users to freely share, modify, and use this Dataset while maintaining this same freedom for others, provided that the original source and author(s) are credited. Ryuichi Yoshioka, Hermann Voglmayr, Akira Hashimoto, Misato Matsumura, Yoshihiro Kudo, Kazuaki Tanaka

Supplementary material 5 *RPB2* alignment dataset used for the first analysis Data type fas Explanation note This dataset is composed of 24 strains of *Splanchospora* newly obtained in this study and 40 members of *Pleosporales* (including 15 members of *Neohendersoniaceae*) and 2 members of *Hysteriales* retrieved from GenBank. For missing *RPB2* sequence data, see Table 1. https://binary.pensoft.net/file/1557645This dataset is made available under the Open Database License (http://opendatacommons.org/licenses/odbl/1.0/). The Open Database License (ODbL) is a license agreement intended to allow users to freely share, modify, and use this Dataset while maintaining this same freedom for others, provided that the original source and author(s) are credited. Ryuichi Yoshioka, Hermann Voglmayr, Akira Hashimoto, Misato Matsumura, Yoshihiro Kudo, Kazuaki Tanaka

Supplementary material 6 *TEF1* alignment dataset used for the first analysis Data type fas Explanation note This dataset is composed of 24 strains of *Splanchospora* newly obtained in this study and 40 members of *Pleosporales* (including 15 members of *Neohendersoniaceae*) and 2 members of *Hysteriales* retrieved from GenBank. For missing *TEF1* sequence data, see Table 1. https://binary.pensoft.net/file/1557646This dataset is made available under the Open Database License (http://opendatacommons.org/licenses/odbl/1.0/). The Open Database License (ODbL) is a license agreement intended to allow users to freely share, modify, and use this Dataset while maintaining this same freedom for others, provided that the original source and author(s) are credited. Ryuichi Yoshioka, Hermann Voglmayr, Akira Hashimoto, Misato Matsumura, Yoshihiro Kudo, Kazuaki Tanaka

Supplementary material 7 ITS alignment dataset used for the second analysis Data type fas Explanation note This dataset is composed of 29 strains of *Splanchospora* newly obtained in this study. For single-gene analysis (Suppl. material 1: fig. S1A), all 29 strains were used, and for combined analysis (Fig. 2), 24 strains were used, excluding cultures RY 152, RY 153, RY 154, RY 158, and RY 161. https://binary.pensoft.net/file/1557647This dataset is made available under the Open Database License (http://opendatacommons.org/licenses/odbl/1.0/). The Open Database License (ODbL) is a license agreement intended to allow users to freely share, modify, and use this Dataset while maintaining this same freedom for others, provided that the original source and author(s) are credited. Ryuichi Yoshioka, Hermann Voglmayr, Akira Hashimoto, Misato Matsumura, Yoshihiro Kudo, Kazuaki Tanaka

Supplementary material 8 *RPB2* alignment dataset used for the second analysis Data type fas Explanation note This dataset is composed of 24 strains of *Splanchospora* newly obtained in this study. https://binary.pensoft.net/file/1557648This dataset is made available under the Open Database License (http://opendatacommons.org/licenses/odbl/1.0/). The Open Database License (ODbL) is a license agreement intended to allow users to freely share, modify, and use this Dataset while maintaining this same freedom for others, provided that the original source and author(s) are credited. Ryuichi Yoshioka, Hermann Voglmayr, Akira Hashimoto, Misato Matsumura, Yoshihiro Kudo, Kazuaki Tanaka

Supplementary material 9 *TEF1* alignment dataset used for the second analysis Data type fas Explanation note This dataset is composed of 24 strains of *Splanchospora* newly obtained in this study. https://binary.pensoft.net/file/1557649This dataset is made available under the Open Database License (http://opendatacommons.org/licenses/odbl/1.0/). The Open Database License (ODbL) is a license agreement intended to allow users to freely share, modify, and use this Dataset while maintaining this same freedom for others, provided that the original source and author(s) are credited. Ryuichi Yoshioka, Hermann Voglmayr, Akira Hashimoto, Misato Matsumura, Yoshihiro Kudo, Kazuaki Tanaka
