# Supplementary material for: Revision of the genus Splanchospora (Pleosporales, Neohendersoniaceae)
Source: IMA Fungus. 2026 Mar 9;17:e179372. doi: 10.3897/imafungus.17.179372 (PMC12993480; doi:10.3897/imafungus.17.179372)
Supplement: Supplementary material 1 — Additional figures and tables [file imafungus-17-e179372-s001.pdf]

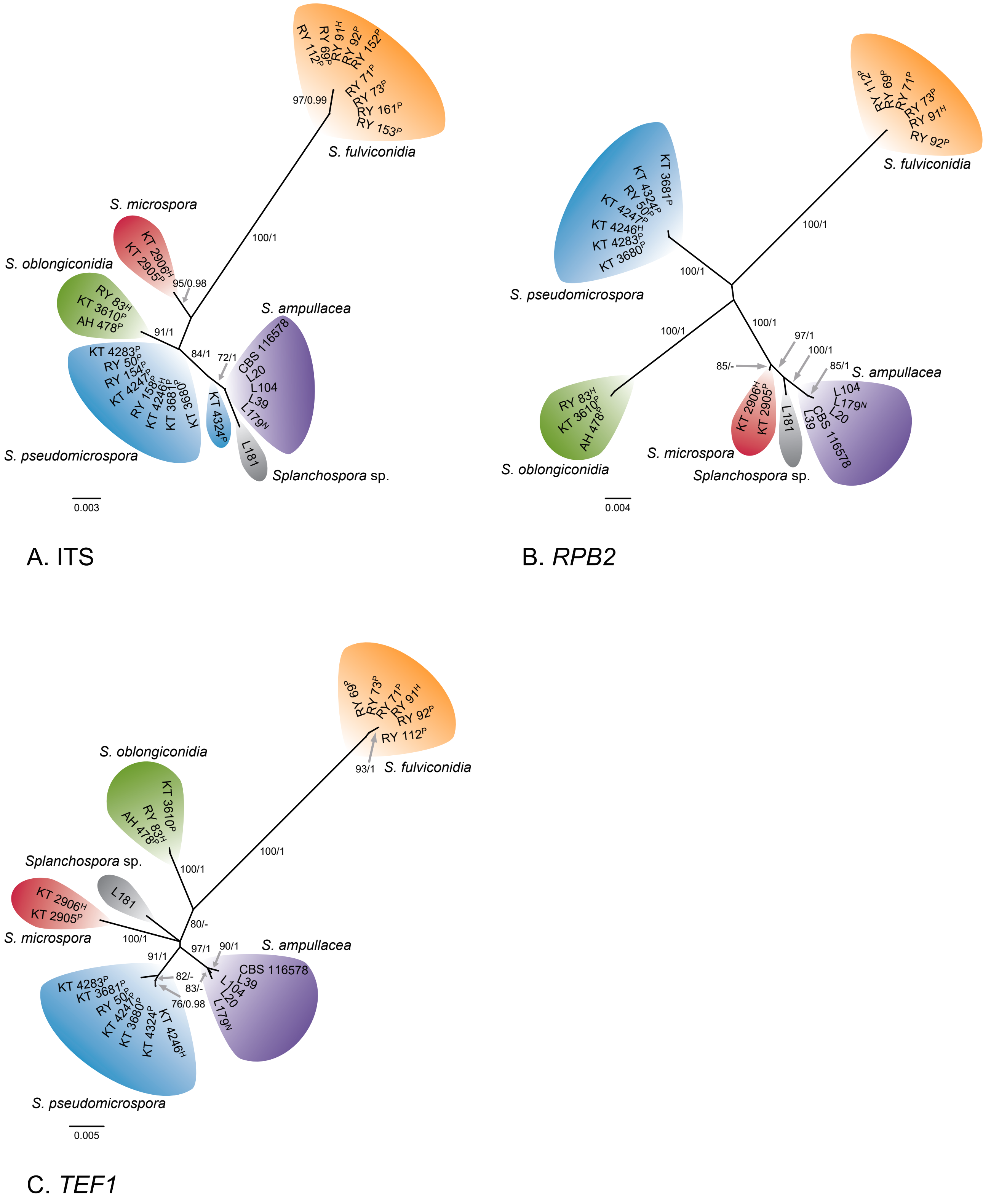

Supplementary Figure S1. Maximum-likelihood (ML) tree of *Splanchospora* based on each ITS (A), *RPB2* (B), and *TEF1* (C). ML bootstrap support (MLBS) values above 70% and Bayesian posterior probability (BPP) values above 0.95 are presented at the nodes as MLBS/BPP. A hyphen ('-') indicates values lower than 70% MLBS or 0.95 BPP. The scale bar represents nucleotide substitutions per site. Ex-holotype, ex-neotype, and ex-paratype strains are indicated as H, N, and P, respectively.

Supplementary Table S1. Specimens and isolates information of *Splanchospora* used in this study.

Taxon	Host†	Country	Original no.	Specimen no.	Type‡	Culture no.	Source of isolation§
Splanchospora ampullacea	1	Sweden	5629c	-		CBS 116578 = UPSC 3376	A
	3	Austria	L20	WU-MYC 0057750 = HHUF 30703		CBS 122932 = culture L20	A
	7	UK	L39	WU-MYC 0057751		CBS 124477 = culture L39	A
	2	Austria	L104	WU-MYC 0057752		culture L104	A
	1	Austria	L179	WU-MYC 0057753 = HHUF 30704	N	CBS 154683 = culture L179	A
S. fulviconidia	4 or 5	Japan	RY 69	HHUF 30705	P	culture RY 69	A
	4 or 5	Japan	RY 71	HHUF 30706	P	culture RY 71	C
	4 or 5	Japan	RY 73	HHUF 30707	P	culture RY 73	A
	4	Japan	RY 91	HHUF 30708	H	MAFF 248153 = culture RY 91	A
	4	Japan	RY 92	HHUF 30709	P	culture RY 92	C
	6	Japan	RY 112	HHUF 30710	P	culture RY 112	C
	4 or 5	Japan	RY 152	HHUF 30711	P	culture RY 152	A
	4 or 5	Japan	RY 153	HHUF 30712	P	culture RY 153	C
	4 or 5	Japan	RY 161	HHUF 30713	P	culture RY 161	A
S. microspora	7	Japan	KT 2905	HHUF 30714	P	culture KT 2905	C
	7	Japan	KT 2906	HHUF 30715	H	MAFF 248150 = culture KT 2906	A
S. oblongiconidia	8	Japan	AH 478	HHUF 30716	P	culture AH 478	A
	9	Japan	KT 3610	HHUF 30717	P	culture KT 3610	C
	5	Japan	RY 83	HHUF 30718	H	MAFF 248152 = culture RY 83	A
S. pseudomicrospora	5 or 3	Japan	KT 3680	HHUF 30719	P	culture KT 3680	A
	5 or 3	Japan	KT 3681	HHUF 30720	P	culture KT 3681	C
	4	Japan	KT 4246	HHUF 30721	H	MAFF 248151 = culture KT 4246	A
	4	Japan	KT 4247	HHUF 30722	P	culture KT 4247	C
	5	Japan	KT 4283	HHUF 30723	P	culture KT 4283	C
	4	Japan	KT 4324	HHUF 30724	P	culture KT 4324	C
	4	Japan	RY 50	HHUF 30725	P	culture RY 50	A
	4 or 5	Japan	RY 154	HHUF 30726	P	culture RY 154	A
	4 or 5	Japan	RY 158	HHUF 30727	P	culture RY 158	A
Splanchospora sp.	1	Austria	L181	WU-MYC 0057754		culture L181	C

† 1 = *Tilia cordata*, 2 = *T.* cf. *cordata*, 3 = *T. platyphyllos*, 4 = *T. japonica*, 5 = *T. maximowicziana*, 6 = *T. kiusiana*, 7 = *Tilia* sp., 8 = *Carpinus cordata*, 9 = *Ostrya japonica*.

‡ H = holotype, N = neotype, P = paratype.

§ A = single ascospore, C = single conidium.

Supplementary Table S2. Primers used to amplify and sequence the small subunit (SSU) of the nuclear ribosomal DNA, the internal transcribed spacer region (ITS), the large subunit (LSU) of the nuclear ribosomal DNA, the RNA polymerase II second largest subunit (*RPB2*) gene, and the translation elongation factor 1-alpha (*TEF1*) gene.

Gene	Primer	Sequence (5'–3')	Direction	Annealing t (°C)	Amplicon length	References
SSU	NS1	GTAGTCATATGCTTGTCTC	forward	42	1.0–1.3 kb	White et al. (1990)
	NS4	CTTCCGTCAATTCCTTTAAG	reverse			White et al. (1990)
	SL1	TGGTTGATCCTGCCAGTA	forward	55	2.0 kb	Landvik et al. (1997)
	NS24mod	GAAACCTTGTTACGACTTTTAC	reverse			Voglmayr and Jaklitsch (2011)
ITS-LSU	V9G	TTAAGTCCCTGCCCTTTGTA	forward	55	2.0 kb	de Hoog and Gerrits van den Ende (1998)
	LR5	TACTTGAAGGAACCCTTACC	reverse			Hopple and Vilgalys (1994)
	LR2R-A†	CAGAGACCGATAGCGCAC	forward			Voglmayr et al. (2012)
	LR3†	CCGTGTTTCAAGACGGG	reverse			Hopple and Vilgalys (1994)
	ITS4†	TCCTCCGCTTATTGATATGC	reverse			White et al. (1990)
ITS	ITS1	TCCGTAGGTGAACCTGCGG	forward	61.5	0.5 kb	White et al. (1990)
	ITS4	TCCTCCGCTTATTGATATGC	reverse			White et al. (1990)
LSU	LR0R	GTACCCGCTGAACTTAAGC	forward	46.2	1.3 kb	Rehner and Samuels (1994)
	LR7	TACTACCACCAAGATCT	reverse			Vilgalys and Hester (1990)
RPB2	dRPB2-5f	GAYACNGAYGAYCGWGAYCAYTTYGG	forward	55	1.2 kb	Voglmayr et al. (2016)
	dRPB2-7r	AANCCCATDGCYTGYTTDCCCAT	reverse			Voglmayr et al. (2016)
	fRPB2-5F	GAYGAYMGWGATCAYTTYGG	forward	55–58	1.1 kb	Liu et al. (1999)
	fRPB2-7cR	CCCATRGCTTGYTTRCCCAT	reverse			Liu et al. (1999)
TEF1	EF1-728F	CATCGAGAAGTTCGAGAAGG	forward	55–58	1.2–1.6 kb	Carbone and Kohn (1999)
	TEF1LLErev	AACTTGCAGGCAATGTGG	reverse			Jaklitsch et al. (2005)
	EF1-2218R	ATGACACCRACRGCRACRGTYTG	reverse			Rehner and Buckley (2005)

† internal primers used only for sequencing.

Supplementary Table S2. (Continued).

References

Carbone I, Kohn LM (1999) A method for designing primer sets for speciation studies in filamentous ascomycetes. Mycologia 91(3): 553–556. https://doi.org/10.1080/00275514.1999.12061051

de Hoog GS, Gerrits van den Ende AHG (1998) Molecular diagnostics of clinical strains of filamentous basidiomycetes. Mycoses 41(5–6): 183–189. https://doi.org/10.1111/j.1439-0507.1998.tb00321.x

Hopple JS, Vilgalys R (1994) Phylogenetic relationships among coprinoid taxa and allies based on data from restriction site mapping of nuclear rDNA. Mycologia 86(1): 96–107. https://doi.org/10.1080/00275514.1994.12026378

Jaklitsch WM, Komon M, Kubicek CP et al. (2005) *Hypocrea voglmayrii* sp. nov. from the Austrian Alps represents a new phylogenetic clade in *Hypocrea*/*Trichoderma*. Mycologia 97(6): 1365–1378. https://doi.org/10.1080/15572536.2006.11832743

Landvik S, Egger KN, Schumacher T (1997) Towards a subordinal classification of the *Pezizales* (*Ascomycota*): phylogenetic analyses of SSU rDNA sequences. Nordic Journal of Botany 17(4): 403–418. https://doi.org/10.1111/j.1756-1051.1997.tb00337.x

Liu YJ, Whelen S, Hall BD (1999) Phylogenetic relationships among ascomycetes: evidence from an RNA polymerase II subunit. Molecular Biology and Evolution 16(12): 1799–1808. https://doi.org/10.1093/oxfordjournals.molbev.a026092

Rehner SA, Samuels GJ (1994) Taxonomy and phylogeny of *Gliocladium* analysed from nuclear large subunit ribosomal DNA sequences. Mycolgical Research 98(6): 625–634. https://doi.org/10.1016/S0953-7562(09)80409-7

Rehner SA, Buckley E (2005) A *Beauveria* phylogeny inferred from nuclear ITS and *EF1-α* sequences: evidence for cryptic diversification and links to *Cordyceps* teleomorphs. Mycologia 97(1): 84–98. https://doi.org/10.1080/15572536.2006.11832842

Vilgalys R, Hester M (1990) Rapid genetic identification and mapping of enzymatically amplified ribosomal DNA from several *Cryptococcus* species. Journal of Bacteriology 172(8): 4238–4246. https://doi.org/10.1128/jb.172.8.4238-4246.1990

Voglmayr H, Jaklitsch WM (2011) Molecular data reveal high host specificity in the phylogenetically isolated genus *Massaria* (*Ascomycota*, *Massariaceae*). Fungal Diversity 46: 133–170. https://doi.org/10.1007/s13225-010-0078-5

Voglmayr H, Rossman AY, Castlebury LA et al. (2012) Multigene phylogeny and taxonomy of the genus *Melanconiella* (*Diaporthales*). Fungal Diversity 57: 1–44. https://doi.org/10.1007/s13225-012-0175-8

Voglmayr H, Akulov OY, Jaklitsch WM (2016) Reassessment of *Allantonectria*, phylogenetic position of *Thyronectroidea*, and *Thyronectria caraganae* sp. nov. Mycological Progress 15: 921–937. https://doi.org/10.1007/s11557-016-1218-4

White TJ, Bruns T, Lee S et al. (1990) Amplification and direct sequencing of fungal ribosomal RNA genes for phylogenetics. In: Innis MA, Gelfand DH, Sninsky JJ, White TJ (Eds) PCR protocols: a guide to methods and applications. Elsevier Academic Press, San Diego, 315–322. https://doi.org/10.1016/B978-0-12-372180-8.50042-1

Supplementary Table S3. Datasets used and statistics resulting from phylogenetic analyses in this study.

Analyses	Total number of characters	Variable sites	Constant sites	Parsimony informative sites	Substitution model	
First analysis (Fig. 1)						
SSU	1,009	232	767	157	GTR+G	
ITS	502	306	176	232	J2ef+G	
LSU	874	270	597	194	GTR+G	
RPB2	1,041	607	434	544	J2+G	1st codon
					J1+G	2nd codon
					J2+G	3rd codon
TEF1 (exon)	747	291	441	228	TVM+G	1st codon
					JC69+G	2nd codon
					J2+G	3rd codon
Total	4,173	1,706	2,415	1,355		
Second multiple-gene analysis (Fig. 2)						
ITS	452	22	430	20	J2ef+G	
RPB2	1,065	88	977	83	TN93+G	1st codon
					F81+H	2nd codon
					TN93+H	3rd codon
TEF1 (intron + exon)	1,253	86	1,167	76	TN93+G	
Total	2,770	196	2,574	179		
Second single-gene analyses (Supplementary Fig. S1)						
ITS	452	22	430	20	K80+H	
RPB2	1,065	88	977	83	TN93+G	1st codon
					F81+H	2nd codon
					TN93+H	3rd codon
TEF1 (intron + exon)	1,253	86	1,167	76	TN93+G	
